# Coherent eddies redistribute microplastics across the water column

**DOI:** 10.1038/s41598-026-60846-7

**Published:** 2026-09-03

**Authors:** Álvaro Cubas, Eugenio Fraile-Nuez, Francisco J. Beron-Vera, Ana Molina-Rodríguez, Francisco Machín, Daura Vega-Moreno

**Affiliations:** 1 Oceanografía Física y Geofísica Aplicada (OFYGA), IU-ECOAQUA, University of Las Palmas de Gran Canaria, Las Palmas, 35017 Spain; 2 Centro Oceanográfico de Canarias, Instituto Español de Oceanografía (IEO), Consejo Superior de Investigaciones Científicas (CSIC), Santa Cruz de Tenerife, Tenerife, 38180 Spain; 3 Department of Atmospheric Sciences, Rosenstiel School of Marine, Atmospheric & Earth Science, University of Miami, Miami, FL 33149 USA; 4 OpenPLAS Group, Chemistry Department, University of Las Palmas de Gran Canaria, Las Palmas de Gran Canaria, 35017 Spain

**Keywords:** Microplastics, Mesoscale eddies, Lagrangian Coherent Structures, Inertial dynamics, Climate sciences, Environmental sciences, Ocean sciences

## Abstract

The ocean interior is emerging as a hidden reservoir of microplastics, yet the mechanisms governing their accumulation below the surface remain poorly understood. Despite growing interest in microplastic distribution throughout the ocean, in-situ observations within smaller-scale oceanographic features such as mesoscale eddies and fronts remain sparse. Here we present in-situ observations of microplastic distributions from 50 to 1200 m within two coherent mesoscale eddies south of the Canary Islands, an anticyclone and a cyclone. Combining physical oceanographic measurements and microplastic sampling with Lagrangian Coherent Structure (LCS) analyses, we show that both eddies enhanced microplastic concentrations in the upper 200 m by up to threefold relative to regional background waters. Small fragments (< 200 μm) dominated microplastic abundances, while fibers were significantly more abundant in the cyclone and larger fragments prevailed in the anticyclonic eddy. These findings reinforce that coherent eddies act as dynamic drivers of microplastic redistribution in the Atlantic Ocean, emphasizing the role of particle-specific inertial dynamics in shaping the fate of plastics throughout the water column.

**Supplementary Information:**

The online version contains supplementary material available at https://doi.org/10.1038/s41598-026-60846-7.

## Introduction

Marine microplastic (MP) pollution has become an omnipresent component of the global ocean, with consequences for marine life and ocean health at multiple levels^1–3^. Despite extensive research on marine microplastics over the past two decades, observational efforts have been largely concentrated in coastal and surface environments, even though the ocean interior comprises most of the ocean volume and likely hosts a substantial fraction of marine microplastics^4–6^. This lack of vertical information limits our understanding of the impacts of microplastics on key oceanic processes, such as the biological carbon pump, which has been observed to be affected by microplastic debris^7–9^. This observational gap is highlighted by the large mismatch between estimated terrestrial plastic inputs to the ocean, on the order of 5–12 million tons per year^10^, and the total amount of plastic observed floating at the sea surface, which is less than 0.3 million tons^11–13^. While this discrepancy has been partly attributed to overestimations of plastic inputs and longer-than-expected surface residence times^14^, delays between coastal inputs and offshore accumulation^15^, incomplete knowledge of key physical drivers, including vertical transport, submesoscale circulation and water mass influence remains a major source of uncertainty^6,16,17^, leaving our understanding of plastic fluxes, pathways, and fate in the ocean far from complete.

Mesoscale eddies are rotating oceanic features that strongly influence lateral transport, mixing, and tracer retention in the open ocean^18^. Over the past decades, different Lagrangian frameworks have further advanced the understanding of eddy-driven transport by identifying materially coherent vortices that resist filamentation and retain fluid over finite time scales^19–23^. Regarding microplastics, recent observations suggest that these structures may condition microplastic distribution even in remote oceanic regions^24^. However, evidence directly linking mesoscale dynamics to microplastic accumulation has so far been largely restricted to surface observations. Brach et al.^25^ reported enhanced concentrations of floating microplastics (> 300 μm) within anticyclonic eddies relative to cyclonic ones, while Nakajima et al.^26^ documented exceptionally high surface concentrations of floating microplastics (> 300 μm) inside an intense cyclonic eddy in the Kuroshio Extension. At smaller scales, Testa et al.^27^ presented in situ observations of subsurface microfiber accumulation within a submesoscale cyclonic vortex, suggesting that such vortices can act as transient reservoirs of microplastics below the surface. More recently, Ding et al.^28^ characterized the distribution of microplastics within mesoscale eddies in the Western North Pacific at the surface and at multiple depths. They reported higher microplastic abundance, size and diversity in anticyclonic eddies in the upper ocean. Despite these advances, in situ observations resolving the internal structure of mesoscale eddies remain scarce, leaving unresolved how these structures may simultaneously transport microplastics and redistribute them vertically within the water column.

Here we address this gap by presenting in situ observations of microplastics concentrations spanning the upper and intermediate ocean (50–1200 m) collected inside two mesoscale eddies of opposite polarity in the Atlantic Ocean. By combining targeted oceanographic and microplastic sampling with Lagrangian diagnostics of material coherence, specifically the Lagrangian-Averaged Vorticity Deviation (LAVD)^20,29^, we investigate how coherent eddy dynamics shape the distribution of microplastics fragments and fibers. This approach allows us to link observed concentration patterns to inertial particle dynamics and to evaluate how mesoscale eddies act as pathways for both lateral transport and vertical redistribution of microplastics within the open ocean.

## Materials and methods

### Study region

The Canary Current System is named after the Canary Current which forms the eastern branch of the North Atlantic subtropical gyre^30,31^. Although in its broadest sense the system includes the upwelling off the western Iberian coast (associated with the Portugal Current)^32^, for this research our focus is on the area comprising the Canary Islands and the northwest of the African coast.

Northeasterly winds drive year-round upwelling-favorable wind stress in the region between 20 and 30 ºN^33,34^, making this one of the four Eastern Boundary Upwelling Systems (EBUS). Moreover, the trade winds interact with the Canary Islands producing airflow diversion to the flanks generating warm wakes in the lee regions where the ocean’s circulation is modified^35–37^.

The ocean currents of the region, like the Canary Current, interact with the Canary Archipelago and the instabilities that develop between Canary Islands and Africa, promote significant mesoscale activity^38–40^. Consequently, a consistent pathway for long-lived eddies, known as the Canary Eddy Corridor, is present^41^ together with upwelling filaments that are quasi-permanent^42–44^.

### Inertial particle dynamics in mesoscale eddies: theoretical background

The adaptation of the Maxey-Riley equation for finite-size particle motion to oceanography^45^ has enabled the derivation of several results relating to the attraction of, or repulsion from, mesoscale vortices of such particles, like plastic debris. When the particles are immersed in the unsteady flow of a fluid, it has been shown that anticyclonic eddies collect heavy particles (i.e., with a density greater than that of the ambient water), while they expel light particles, and vice versa for cyclonic eddies^20,46^. If the particles float at the ocean surface, it has been demonstrated that anticyclonic eddies behave as finite-time attractors for them, while cyclonic eddies act as finite-time repellers^47^, explaining the results obtained by Brach et al.^25^. Finally, when the particles interact elastically, according to Hooke’s law^48^ or a more general nonlinear law^49^, which is needed to model the motion of *Sargassum* seaweed, anticyclonic eddies always trap such systems of interacting particles, while cyclonic eddies trap them when the particles’ interaction is sufficiently strong.

More recently, Pratt and Rypina^50^ discussed the existence of closed-loop attractors for slightly buoyant particles within a steady three-dimensional vortex. In this idealized setting, an axisymmetric circulation foliates the flow into a family of nested flow-invariant surfaces, within whose inner regions, a single particle attractor may exist, whose position depends on the density and size of the particle. When non-axisymmetric perturbations are introduced, which may also be quasiperiodic in time, additional attractors can emerge.

### In situ hydrographic and microplastic observations

**Fig. 1:**
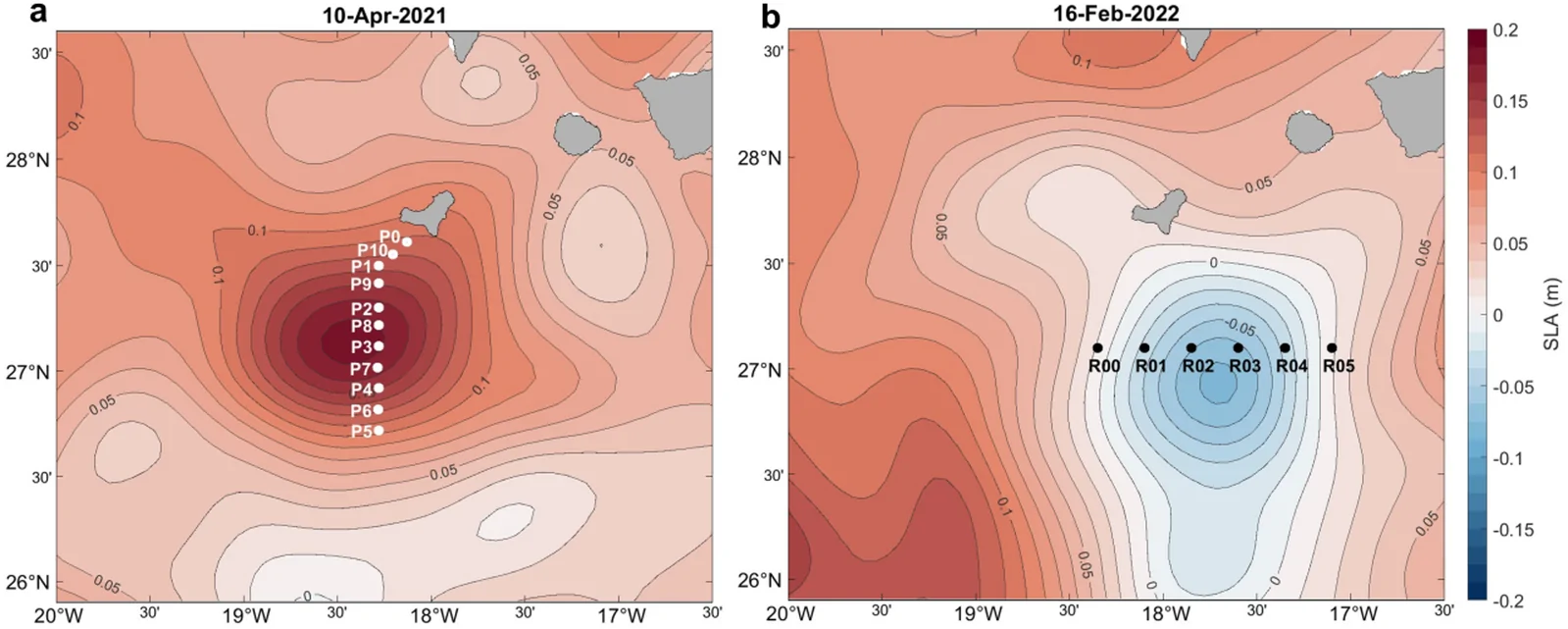
Map of sampling stations overlaid on Sea Level Anomaly (SLA) contours for (a) the anticyclonic eddy and (b) the cyclonic eddy. SLA data are retrieved from the Copernicus Marine Service product “European Seas Gridded L4 Sea Surface Heights and Derived Variables Reprocessed 1993 Ongoing” (dataset ID: cmems_obs-sl_eur_phy-ssh_my_allsat-l4-duacs-0.0625deg_P1D_202411; https://doi.org/10.48670/moi-00141, accessed 10 March 2025).

During two oceanographic cruises conducted south of El Hierro Island (Canary Islands, Spain), two mesoscale eddies were sampled. The first eddy, exhibiting surface anticyclonic circulation as indicated by altimetry data, was surveyed along a quasi-meridional transect of eleven stations (P0–P10) spanning approximately 27 h between April 10th and 11th, 2021, during the VULCANA-III-0421 cruise (Fig. 1A). The second eddy, characterized by surface cyclonic circulation, was sampled on February 16th, 2022, along a zonal transect of six stations (R00–R05) over approximately 14 h during the VULCANA-III-0222 cruise (Fig. 1B). Both cruises were conducted on board the R/V Ángeles Alvariño as part of the VULCANA-III Project, led by the Spanish Institute of Oceanography (IEO-CSIC).

On the one hand, the oceanographic data were obtained through a SeaBird 911 Plus CTD with added sensors installed in a rosette in order to measure temperature, conductivity, pressure, dissolved oxygen, pH, turbidity and fluorescence. These data were then used to calculate derived variables such as conservative temperature, absolute salinity, potential density anomaly ($\:{\sigma\:}_{\theta\:}$), geostrophic velocity, Brunt-Väisälä frequency, and Apparent Oxygen Utilization (AOU). These parameters were calculated using the TEOS-10 equation through the Gibbs-SeaWater (GSW) Oceanographic Toolbox^51^ in MATLAB R2023a. To compute the geostrophic velocity, we use as reference the geostrophic speed at the pixel closest to the CTD Stations at 5 m depth from the Copernicus Marine Service product “Multi Observation Global Ocean 3D Temperature Salinity Height Geostrophic Current and MLD” (dataset ID: dataset-armor-3d-rep-weekly_202012; https://doi.org/10.48670/moi-00052, accessed 24 March 2025), which was interpolated from weekly to daily resolution using cubic spline interpolation. In addition, anomalies of conservative temperature, absolute salinity, $\:{\sigma\:}_{\theta\:}$ and dissolved oxygen were computed by subtracting the regional [15.5ºW, 20ºW] $\:\times\:$ [26ºN, 29ºN] monthly means given by the World Ocean Atlas 2023 (WOA 2023) dataset^52^ from the CTD-derived values throughout the water column.

On the other hand, for the MPs sampling, seawater was collected with a 24-bottle Niskin rosette using 5–6 bottles (60–72 L) per sample. Thus, for each station 4 sampling depths were considered ranging from 50 to 1200 m. The sampling depths were selected to represent key water column features, from the base of the mixed layer through central and intermediate waters, within the constraints of available ship time. Immediately after the bottle closure and recovery of the rosette, water from each selected depth was filtered through transparent and flexible tubes to a collector with a 100 μm mesh. The retained material was rinsed into a 47 mm Whatman GF/F filter held in a filtration pump and then transferred to Petri dishes and stored at -20 °C until analysis. In total, 44 samples were obtained from the anticyclonic eddy (however to avoid the possible coastal effects the stations P0 and P10 are omitted) and 27 from the cyclonic eddy. Blank controls were processed onboard to assess potential procedural contamination.

Microplastic particles were counted visually under stereomicroscopes. Detected MPs were classified into three categories: small fragments (< 200 μm), large fragments (> 200 μm) and fibers. The concentrations at each depth were calculated considering the counted particles and the volume of water filtered. It should be noted that both fragment fractions despite their names are commonly categorized as small microplastic (SMPs) due to their size below 1000 μm^53^. Moreover, the chemical composition of the collected samples was studied by Rodríguez et al.^54^.

### Assessment of material coherence

To assess the material coherence of the sampled eddies, we apply, as anticipated, the LAVD methodology^20^. Eddies detected using this technique have the property that each element along their boundaries rotates by the same amount over the material assessment period, say $\:[{t}_{0},{t}_{0}+T]$. This property prevents their boundaries from experiencing breakaway filamentation, thereby qualifying as LCSs of elliptic type, coherent rotating structures that act as finite-time barriers to fluid transport, isolating their interior from the surrounding ocean. As discussed in Sect. 2.2, this barrier property is central to understanding how such structures can selectively trap or expel inertial particles, potentially including microplastics depending on their buoyancy and the eddy polarity. The time-$\:{t}_{0}$ position of the material boundaries of such rotationally coherent eddies (RCEs) is specifically given by the outermost convex level sets of the LAVD field, which computed over $\:[{t}_{0},{t}_{0}+T]$, enclose the relative maxima of the field.

The vorticity involved in the computation of the LAVD field was estimated using daily geostrophic velocity data provided by the Copernicus Marine Service product, European Seas Gridded L4 Sea Surface Heights, and Derived Variables Reprocessed 1993 Ongoing. The velocity field was spatially interpolated using a cubic spline method prior to trajectory integration. Trajectory integrations required to evaluate Lagrangian averages were carried out using a 4th-order Runge-Kutta scheme with a time step of 0.1 days with cubic interpolation in space and time. Convergence of the trajectories was verified by confirming that reducing the time step did not produce significant changes in the resulting RCE boundaries. The computational grid was configured to cover the domain [21ºW, 12ºW] $\:\times\:$ [24ºN, 30ºN] with a spacing of 0.05º, and an auxiliary grid with a spacing ratio of 0.1 relative to the computational grid was used for the numerical estimation of velocity gradients. A very small convexity deficiency of $\:{10}^{-4}$ was permitted in determining the boundaries of the detected RCEs.

The anticyclonic RCE was detected on $\:{t}_{0}=$ March 10, 2021 (one month before sampling) from a computation with * T=40* days. In contrast, the cyclonic RCE was detected on $\:{t}_{0}=$ February 7, 2022 (9 days before the sampling) from a computation with * T=20* days. In each case, t₀ was placed so as to maximise the pre-sampling coverage of the integration window, since only the flow history preceding the in-situ measurements can have shaped the observed microplastic distribution; the window was nevertheless extended modestly beyond the sampling date (~ 10 days in both cases) so as to verify that the identified material boundary remained coherent through, and not merely up to, the sampling event.

### Relative retention parameter

Furthermore, we applied the relative retention parameter ($\:{\mathrm{R}}_{\mathrm{r}}$) to quantitatively assess the degree of microplastic enrichment within eddies relative to surrounding waters based on in situ observations:

1 $$\:{\mathrm{R}}_{\mathrm{r}}=\frac{M{P}_{eddy}-M{P}_{outside}}{M{P}_{outside}},$$

where $\:M{P}_{eddy}$ represents the average MP concentration within the eddy, and $\:M{P}_{outside}$ corresponds to the average concentration outside the eddy. Therefore, a positive value indicates accumulation of MPs. We do not use as reference value the mean concentration of the stations outside the detected RCE. This is because they are placed in the periphery of the eddy where its SLA signal can be observed, thus, the microplastic concentration may not be representative for the area. Ideally, one or more stations sufficiently distant from both eddies would have provided a more robust regional background; however, the available ship time limited such sampling. Combined with the patchy and temporally variable nature of microplastic distributions in the ocean, this introduces some uncertainty in the $\:{\mathrm{R}}_{\mathrm{r}}$estimates, which should be interpreted accordingly.

Therefore, $\:{\mathrm{R}}_{\mathrm{r}}$ was estimated in the upper 200 m of the water column for small fragments and fibers using the reference values of the region from Vega-Moreno et al.^55^: 20.3 items/L for fragments < 500 μm and 2.2 items/L for fibers. These values were collected across a wider spatial and temporal domain within the region, reducing the likelihood of systematic mesoscale influence, and their sum (22.5 items/L) is comparable to the mean concentration for MPs sized 32–651 μm in the upper 200 m of the Atlantic Ocean (22 items/L) reported by Zhao et al.^6^ supporting their use as a regional background estimate. It can be observed that the relative retention for small fragments is going to be underestimated as the reference upper bound of the size fraction differs from the one used in this study. However, as small fragments (< 200 μm) are the most abundant (Sect. 3) the uncertainty can be considered acceptable. The opposite takes place for large fragments (> 200 μm), thus, its estimation would involve a greater error.

## Results

### Material coherence

**Fig. 2:**
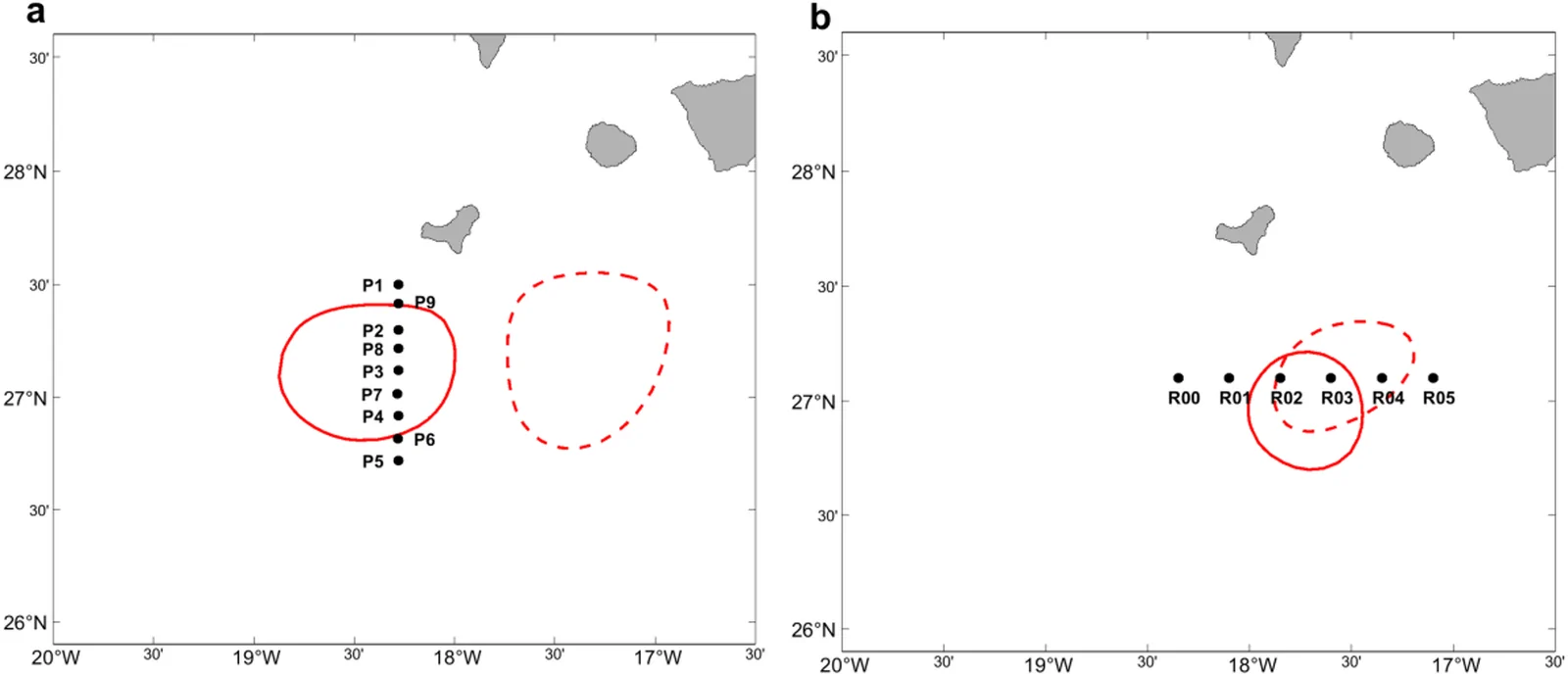
Detected RCEs for the (a) anticyclonic eddy and (b) cyclonic eddy superimposed with the sampling stations. The dashed red contour represents the boundary at time $\:{\boldsymbol{t}}_{0}$ (March 10th, 2021 for the anticyclonic eddy; February 7th, 2022 for the cyclonic eddy). The bold contour shows the advected initial boundary to the sampling time. For the anticyclonic eddy boundary an elapsed time of 40 days was used while 20 days was utilized for the cyclone.

Both eddies were detected as RCEs during time intervals that encompassed the in situ sampling period, with several sampling stations located within their boundaries (Fig. 2). In the case of the anticyclone, stations P1 and P5 lay outside the contour, P9 and P6 are positioned very close to the boundary, and the remaining stations were located inside it. For the cyclone, only stations R02 and R03 were situated within the boundary, although all stations were aligned with the SLA signal (Fig. 1).

Furthermore, the anticyclone was identified as a RCE over a 40-day period, beginning approximately one month prior to the sampling, whereas the cyclone was detected over a 20-day period starting nine days before the sampling. Consequently, the anticyclone maintained material coherence over a longer period (40 days) compared to the cyclone (20 days), implying a more persistent barrier to fluid exchange at the time of sampling.

### Main oceanographic characteristics of the eddies

#### Anticyclonic eddy

**Fig. 3:**
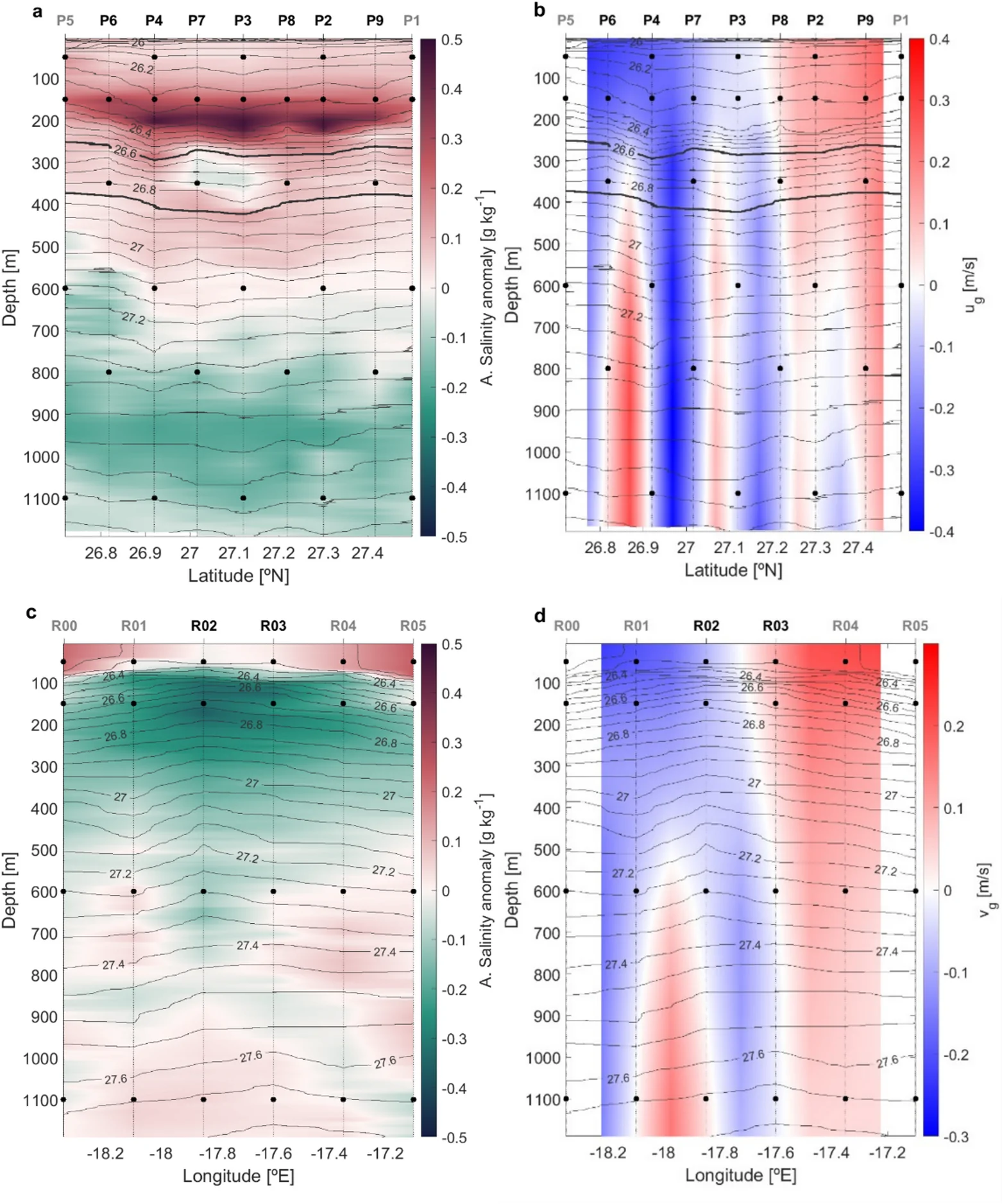
Vertical sections of (a, c) absolute salinity anomaly and (b, d) geostrophic velocity superimposed with the isopycnals for the (a, b) anticyclonic and (c, d) cyclonic eddies. Bold lines in (a) and (b) represent the 26.85 $\:\boldsymbol{k}\boldsymbol{g}\:{\boldsymbol{m}}^{-3}$ and 26.6 $\:\boldsymbol{k}\boldsymbol{g}\:{\boldsymbol{m}}^{-3}$ isopycnals. For the anticyclonic eddy (b), positive (red) and negative (blue) zonal geostrophic velocity values indicate flow out of and into the page, respectively. For the cyclonic eddy (d), positive (red) and negative (blue) meridional geostrophic velocity values indicate flow into and out of the page, respectively.

Figure 3A presents the meridional transect (stations P1-P9) of absolute salinity along with its anomaly superimposed with the isopycnals. These sections reveal the classic dome- and bowl-shaped isopleths characteristic of an intrathermocline eddy. The eddy core is located at approximately 350 m depth, in the lower part of the pycnocline, and is marked by negative anomalies of absolute salinity (Fig. 3A) and dissolved oxygen (Supplementary Figure S4). Above it, a distinct subsurface water lens is observed, characterized by positive anomalies in absolute salinity. This layer also exhibits positive anomalies in conservative temperature and dissolved oxygen, together with a relative weakening of stratification (Supplementary Figures S1, S4 and S5).

Regarding the zonal geostrophic velocity (Fig. 3B), the anticyclonic eddy exhibits a well-defined anticyclonic circulation above the pycnocline, with velocity values ranging from approximately − 0.3 m/s at the southern stations to + 0.2 m/s at the northern stations. Below the pycnocline, within and around the eddy’s subsurface core, the velocity field becomes more complex, displaying alternating cyclonic and anticyclonic cells. This pattern likely reflects temporal variability and measurement delays during the survey. Despite these local structures, the overall circulation remains predominantly anticyclonic throughout the eddy’s vertical structure.

#### Cyclonic eddy

Figure 3C displays the zonal sections of absolute salinity and its anomaly for the cyclonic eddy. A pronounced upward doming of the isopleths is evident, especially at station R02, indicative of upwelling within the eddy. At the base of the pycnocline, marked by a peak in Brunt-Väisälä frequency, the eddy exhibits its largest anomalies: -0.3 $\:\mathrm{k}\mathrm{g}/{\mathrm{m}}^{3}$ in potential density, -2.0 °C deviation in conservative temperature (Supplementary Figure S9), and + 0.4 g/kg in absolute salinity. This sharp pycnocline separates the mixed layer, characterized by opposite-sign anomalies of these variables, from the eddy’s interior.

The meridional geostrophic velocities (Fig. 3D) reveal a clear cyclonic circulation extending throughout the entire water column. Velocity values range from approximately − 0.2 m/s to + 0.2 m/s at the eddy’s periphery.

### Microplastic distribution

#### Differences and similarities between eddies in microplastic concentrations

Table 1 summarizes statistical parameters for microplastic concentrations inside and outside the coherent contour observed in both eddies. The stations P6 and P9 of the anticyclonic eddy are omitted to reduce uncertainties as well as the samples collected below 1000 m where the influence of the eddy can be negligible. After these exclusions, the cyclonic and anticyclonic eddies were represented by *n* = 6 and *n* = 15 data points, respectively. Differences between eddies were evaluated using two-sided Welch’s t-tests, selected for their robustness to unequal variances and small sample sizes, and were complemented by non-parametric Mann–Whitney tests to assess sensitivity to distributional assumptions. Fiber concentrations were significantly higher in the cyclonic eddy (Welch: t(5.71) = 4.07, *p* = 0.0073; Mann–Whitney: *p* = 0.0021). In contrast, large fragments were significantly more abundant in the anticyclonic eddy (Welch: t(14.19) = − 3.86, *p* = 0.0017; Mann–Whitney: *p* = 0.0005). For small fragments, neither test indicated a significant difference between eddies (Welch: t(18.97) = − 0.77, *p* = 0.4526; Mann–Whitney: *p* = 0.9690), indicating comparable concentrations across both systems.

Moreover, the data reveal that small fragments constitute the dominant fraction of the microplastic counting in both eddies. In terms of secondary dominance, fibers are more abundant than large fragments in the cyclonic eddy, while in the anticyclonic eddy, the opposite pattern is observed.

Using the same statistical framework, concentrations were subsequently compared inside and outside the coherent boundaries of each eddy. The anticyclonic eddy was represented by *n* = 15 inside and *n* = 6 outside data points, while the cyclonic eddy comprised *n* = 6 inside and *n* = 12 outside data points. In the anticyclonic eddy, fiber concentrations were significantly higher outside the coherent contour (t(6.23) = 2.63, *p* = 0.0376), a result that was also supported by the Mann–Whitney test (*p* = 0.0127). In the cyclonic eddy, outside–inside contrasts were detected for fragment-type microplastics using Welch’s t-tests, with higher concentrations outside the coherent contour for both large fragments (t(15.58) = 2.20, *p* = 0.0436) and small fragments (t(13.96) = 2.26, *p* = 0.0402). However, these latter contrasts were sensitive to the choice of statistical test and were not consistently supported by non-parametric analyses (Mann-Whitney: large fragments *p* = 0.0750; small fragments *p* = 0.0546). All remaining comparisons did not show statistically significant differences and are reported in Supplementary Table S1.

**Table 1:** Mean, standard deviation (std), minimum and maximum concentration of fibers, small fragments and large fragments in the anticyclonic and cyclonic eddies using data from stations outside and inside the coherent boundary. Data below 1000 m are omitted. For the anticyclone, stations P6 and P9 are also omitted due to being located very close to the contour.

	Anticyclonic eddy				Cyclonic eddy			
	Inside		Outside		Inside		Outside	
	Mean (std)	Min Max	Mean (std)	Min Max	Mean (std)	Min Max	Mean (std)	Min Max
Fibers [items/L]	2.7 (0.7)	1.8 4.0	4.2 (1.3)	3.0 6.2	5.7 (1.7)	2.9 7.6	6.4 (3.3)	2.6 16.0
Small fragments (< 200 μm) [items/L]	53.0 (41.0)	12.7 167.4	60.6 (40.4)	33.7 142.0	43.4 (16.2)	23.3 67.5	64.8 (23.3)	41.9 130.9
Large fragments (> 200 μm) [items/L]	5.8 (5.2)	1.9 20.3	4.6 (2.4)	2.1 9.1	0.6 (0.3)	0.3 1.0	1.0 (0.5)	0.4 1.9

Regarding the relative retention of microplastics in the upper 200 m, the anticyclonic eddy exhibits a value for small fragments of 2.3 while 1.0 was obtained for the cyclone. These values indicate that the concentrations of small fragments within both eddies in the upper 200 m were approximately between two and three times higher (230% and 100% respectively) than the average of the region. In the case of fibers, the $\:{R}_{r}$ resulted in 0.4 for the anticyclonic eddy and 1.6 for the cyclonic eddy, corresponding to increases of 40% and 160% respectively.

#### Anticyclonic eddy

**Fig. 4:**
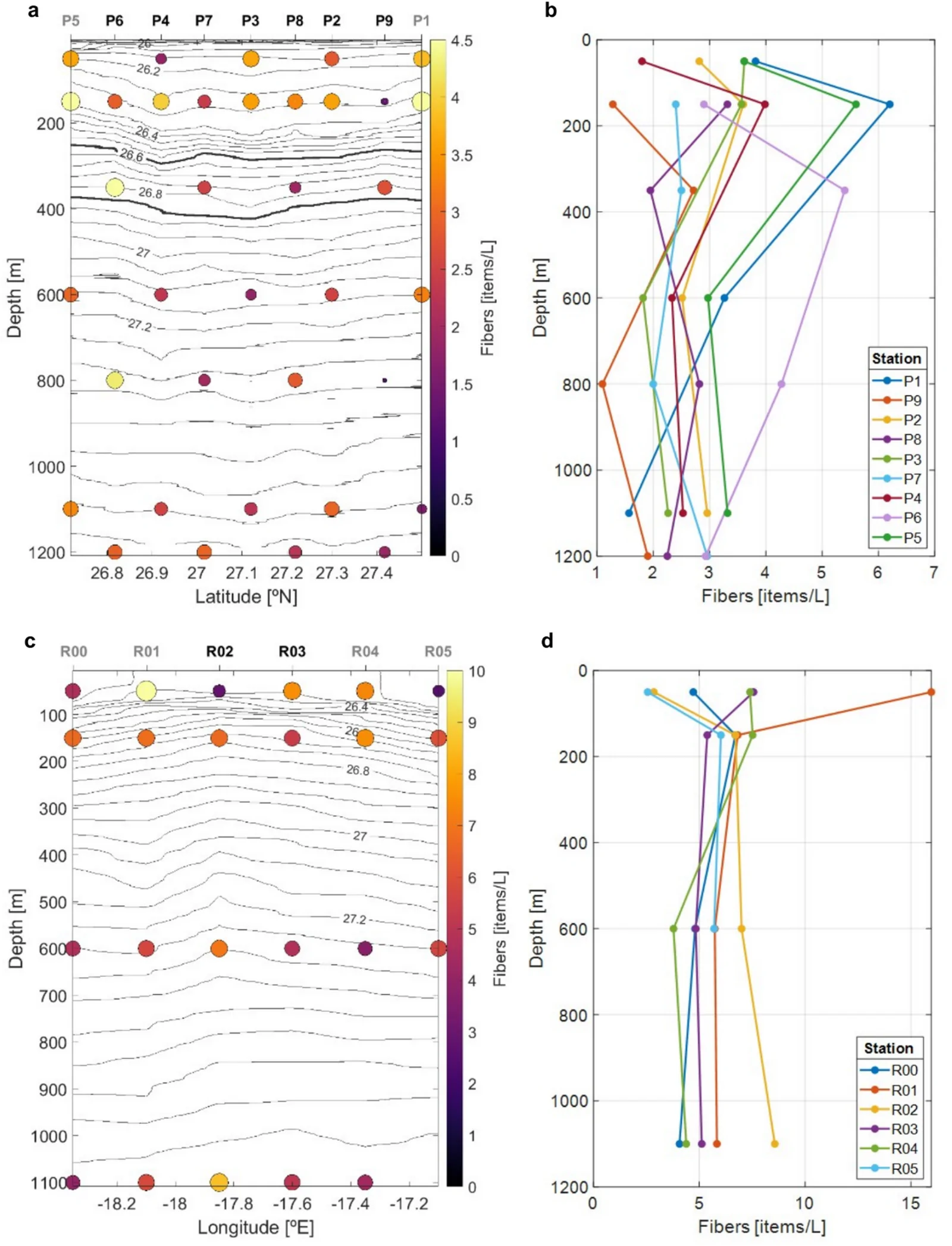
Representation of (a, c) vertical section of fibers concentration superimposed with the isopycnals and (b, d) the vertical profiles for each station for the (a, b) anticyclonic and (c, d) cyclonic eddies. The bold lines in (a) represent the 26.85 $\:\boldsymbol{k}\boldsymbol{g}\:{\boldsymbol{m}}^{-3}$ and 26.6 $\:\boldsymbol{k}\boldsymbol{g}\:{\boldsymbol{m}}^{-3}$ isopycnals. Stations outside the coherent boundary (label in gray color) are also considered.

Starting with the microplastic distribution, in the case of the anticyclonic eddy, Fig. 4 reveals that the highest fiber abundances occur within the mixed layer above the pycnocline (< 200 m), peaking at 150 m depth. Interestingly, fiber concentration peaks in the stations located outside the RCE. Another feature is a minimum that can be found around 600 m. Within the eddy core itself, fiber levels are similar to the surrounding waters, but between 50 and 800 m the stations outside the coherent boundary are in better agreement with Table 1. Below 200 m, fiber counts generally decrease with depth at all stations, until stabilizing at 1100 m depth.

**Fig. 5:**
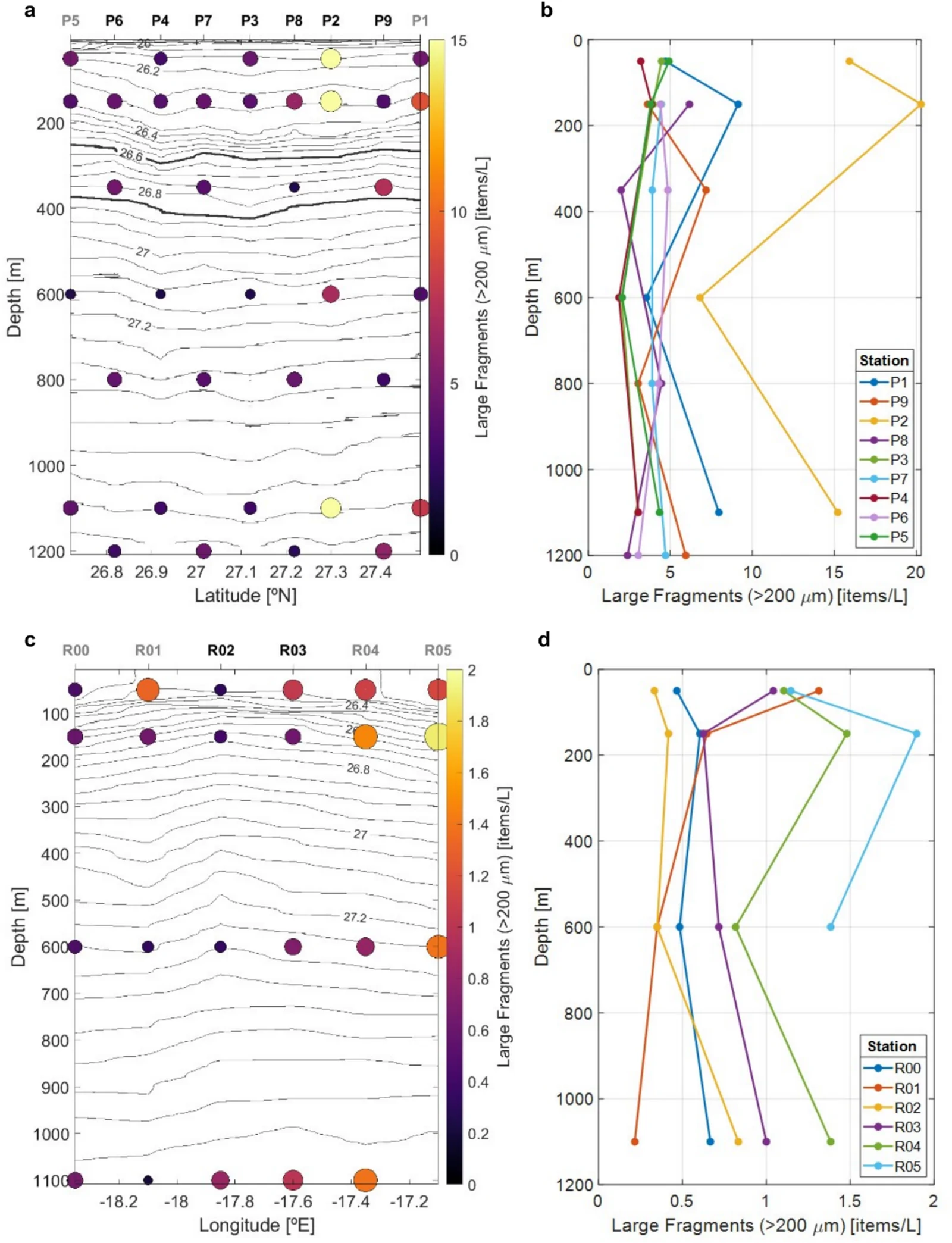
Representation of (a, c) vertical section of large fragments concentration superimposed with the isopycnals and (b, d) the vertical profiles for each station for the (a, b) anticyclonic and (c, d) cyclonic eddies. The bold lines in (a) represent the 26.85 $\:\boldsymbol{k}\boldsymbol{g}\:{\boldsymbol{m}}^{-3}$ and 26.6 $\:\boldsymbol{k}\boldsymbol{g}\:{\boldsymbol{m}}^{-3}$ isopycnals. Stations outside the coherent boundary (label in gray color) are also considered.

Regarding large fragments, elevated values can be seen at station P2 compared to the other profiles (Fig. 5). Horizontally, fragments tend to accumulate at the northernmost stations (P1, P2 and P9), closest to the coast of El Hierro. Vertically, most stations show relatively uniform fragment counts, except at P1, P2 and P9, which display a maximum between 150 and 400 m, a minimum from 600 to 800 m, and a second maximum below 1000 m.

**Fig. 6:**
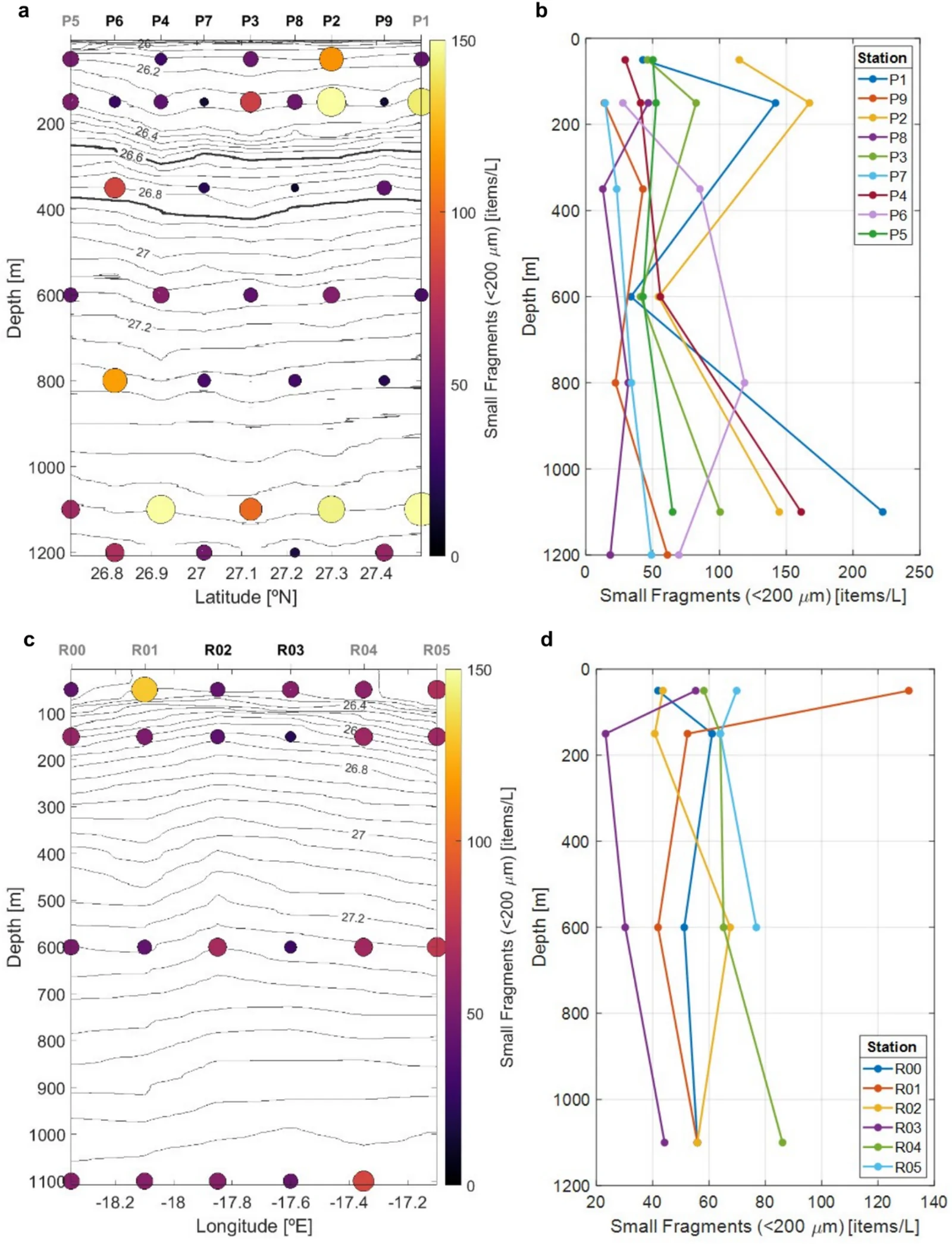
Representation of (a, c) vertical section of small fragments concentration superimposed with the isopycnals and (b, d) the vertical profiles for each station for the (a, b) anticyclonic and (c, d) cyclonic eddies. The bold lines in (a) represent the 26.85 $\:\boldsymbol{k}\boldsymbol{g}\:{\boldsymbol{m}}^{-3}$ and 26.6 $\:\boldsymbol{k}\boldsymbol{g}\:{\boldsymbol{m}}^{-3}$ isopycnals. Stations outside the coherent boundary (label in gray color) are also considered.

The vertical distribution of small fragments mimics that of fibers, with elevated concentrations above the pycnocline and close to the eddy borders (Fig. 6). Furthermore, the distribution presents a shared minimum at 600 m (Fig. 6). In the mixed layer, the northernmost stations exhibit higher concentrations compared to the rest. Notably, stations P5, P4, P3, P2 and P1 show a pronounced maximum at 1100 m depth, levels comparable to or exceeding those in the mixed layer (Fig. 6). It is also remarkable that within the core, at 350 m depth there is a minimum of microplastics that can be observed in stations P7 and P8. Additionally, station P2 again stands out with higher concentrations, though the contrast with other stations is less pronounced than for large fragments (Fig. 6). The cause of these elevated concentrations at P2, common to both fragment classes but absent in fibers, cannot be identified with certainty from the present dataset, although generic contamination, which would be expected to affect fibers as well, appears unlikely.

#### Cyclonic eddy

As shown in Fig. 4, the horizontal distribution reveals slightly higher concentrations of fibers below the pycnocline at R02, although the previous statistical test showed no differences between the inner and outer sections of the eddy. Vertically, fibers are fairly homogeneous beneath the pycnocline ($\:\sim$100 m), although a local minimum is observed at 50 m depth, except at stations R01 and R03, where fiber counts peak instead. Interestingly, a secondary minimum at 600 m depth, previously observed in the anticyclonic eddy, reappears here, especially pronounced at stations R04 and R01.

Horizontally, large fragments tend to accumulate in the eastern part of the eddy, particularly at stations R04 and R05 (Fig. 5). Moreover, between 50 and 600 m R02 shows minimal concentrations. Vertically, most stations exhibit higher concentrations in the upper layers (50–150 m), peaking at 150 m. Exceptions are found at stations R01 and R03, where maximum values occur closer to 50 m. Similar to the other MP categories, a minimum is detected at 600 m across all stations except R01, where concentrations decline with depth (Fig. 5).

Small fragments tend to concentrate at the easternmost stations (R04 and R05), although station R03 presents a noticeable minimum (Fig. 6). Vertically, the 600 m minimum appears again, albeit less clearly than in the anticyclonic eddy, and the overall distribution below the pycnocline remains relatively homogeneous. Above the pycnocline, however, there is more variability with maxima observed at stations R01, R02, R03 and R05, while stations R00 and R04 show minimum values in this upper layer (Fig. 6).

Additionally, in Figs. 4, 5 and 6 the sample obtained at 50 m in R01 highlights a common maximum for all the microplastic categories studied.

## Discussion

### Main oceanographic features of the eddies and material coherence

Regarding the eddy sampled in April 2021, the in situ data suggest that it was an intrathermocline eddy, as evidenced by the bowl- and dome-shaped isopleths characteristic of such features (e.g., Barceló-Llull et al.^56^; Dugan et al.^57^. The trapping of low-oxygen waters associated with upwelling, together with the remineralization of organic matter, could explain the observed negative oxygen anomaly in the subsurface core.

In terms of material coherence, the anticyclonic eddy can be classified as a Lagrangian coherent vortex over a 40-day period starting on March 10, 2021, approximately one month before the sampling (Fig. 2). This is consistent with the eddy trapping and transporting water parcels and attracting floating inertial particles during this interval, at least at the surface, as predicted by Beron-Vera et al.^47^. Interestingly, the relatively weakly stratified, high-oxygen, warmer, and saltier water lens observed above the pycnocline appears to be horizontally bounded by the coherent boundary. This type of structure is consistent with submesoscale subduction processes previously reported in anticyclonic eddies^58,59^. Its apparent relationship with the detected RCE suggests that such subduction phenomena may be confined within coherent eddies, although further studies are required to confirm this link.

For the cyclonic eddy, the material coherence analysis indicates similar behaviour, but over a shorter period beginning just nine days before the sampling. Consequently, at the time of observation, the eddy has had less time to act as a finite-time attractor or repeller of inertial particles, and the particle-specific redistribution effects associated with material coherence are therefore expected to be less pronounced in the cyclonic eddy than in the anticyclonic eddy. Additionally, as station R02 displays a relatively prominent upwelling signature in the isopleths, it can be inferred that it was located closer to the coherent vortex centre across depths, where such processes tend to occur in cyclonic coherent vortices^60^.

Material coherence throughout the water column could not be directly evaluated due to the lack of high-resolution velocity data at depth capable of resolving these dynamics for the sampled eddies. Nevertheless, rotationally coherent eddies typically exhibit a vertically conical structure^60,61^, providing a basis for extending the surface coherence detected here to depth. Accordingly, the sampled eddies may have trapped water parcels across multiple depths and influenced the trajectories of submerged inertial particles, in agreement with the theorem of Haller et al.^20^.

### Impact of eddy dynamics on microplastic distribution

As expected from the fragmentation of larger plastic items, small plastic fragments were the most abundant microplastic category in both eddies, consistent with observations from the open ocean^62–64^. The accumulation of large fragments at the northern stations of the anticyclonic eddy and at the eastern stations of the cyclonic eddy, observed for both large and small fragments, may be linked to their proximity to the coast of El Hierro, where microplastic concentrations tend to be higher^6^. These regions may also correspond to lee areas associated with island-wake effects, which can modify local circulation and generate low-speed flow regions that, together with other dynamical factors, may favour microplastic accumulation^3,6,65,66^. In addition, the preferential accumulation at the northern stations of the anticyclonic eddy may be related to eddy asymmetries, previously documented in similar settings (e.g., Barceló-Llull et al.^56^. In this eddy, geostrophic velocities at the northern stations were lower than those at the southern ones, consistent with such asymmetries. Comparable asymmetries may also exist in the cyclonic eddy but were not detected here.

The $\:{R}_{r}$ values further illustrate the contrasting behaviour between eddies. The cyclonic eddy exhibited higher retention for fibers (1.6) than for small fragments (1.0) within the upper 200 m, whereas the anticyclonic eddy showed the opposite trend, with higher retention for small fragments (2.3) and a much lower occupation by fibers (0.4). These differences reveal particle-specific dynamics within mesoscale eddies, suggesting that anticyclonic RCEs tend to accumulate fragments, while cyclonic RCEs are more prone to retain fibers, at least in the upper layers (< 200 m). In both cases, positive $\:{R}_{r}$ values indicate net retention relative to previous regional studies.

Notably, fibers were significantly less abundant inside the RCE boundary of the anticyclonic eddy, whereas the cyclonic eddy, particularly at station R02, tended to accumulate them. This preferential association of fibers with cyclonic rotation is consistent with recent submesoscale observations reported by Testa et al.^27^. Although their analysis focused on a submesoscale vortex and therefore a different dynamical regime, both studies suggest that cyclonic vortices tend to retain fibers. Furthermore, Testa et al.^27^ reported enhanced microfiber concentrations near the eddy edge, defined there through hydrological rather than Lagrangian coherence criteria, a pattern that closely mirrors the microplastic abundances observed along the boundaries of the rotationally coherent eddies sampled in this study.

The prominent accumulation of microplastics near the peripheries of the RCEs aligns with the general theoretical expectation that the boundaries of these vortices act as transport barriers for fluid parcels and near-neutrally buoyant inertial particles^20^, constraining exchange with surrounding waters. This suggests that most sampled microplastics behave as near-neutrally buoyant particles, implying that these RCEs do not generally act as persistent attractors or repellers of microplastics below the surface.

Nevertheless, deviations from neutral buoyancy and shape-induced inertial effects may still give rise to different behaviours. For instance, the apparent repelling tendency of fibers in the anticyclonic eddy and their accumulation within the cyclone are suggestive of the predictions for positively buoyant particles^20^, although the theorem on which such predictions are based assumes rigid spherical particles. Similarly, the enhanced retention of fragments, particularly large fragments, in the anticyclonic eddy may reflect slight inertial biases that allow certain particle types to remain closer to, or cross, the coherent boundary. While these mechanisms do not overturn the dominant barrier effect, they likely modulate the degree to which different microplastic categories occupy the interior versus the periphery of each eddy. Notably, eddy edges are likely strain-dominated regions, where slightly negatively buoyant particles tend to accumulate^66^, a behaviour consistent with the enhanced fragment concentrations observed near the boundaries of the eddies, suggesting that fragments may behave as slightly negatively buoyant particles. In addition, non-axisymmetric perturbations, likely associated with time-dependent instabilities can give rise to additional attractors for slightly buoyant particles in three-dimensional eddy-flows, with dense particles being repelled from these same features^50,66^. However, given the long attraction timescales associated with such features and the limited evidence for subsurface ring attractors in oceanic settings, it is more likely that particles circulate within a chaotic regime between potential attractors^66^, which may nonetheless contribute to the observed spatial variability in microplastic concentrations.

The lack of statistical significance in some inner-outer contrasts does not constitute evidence against the proposed framework but is compatible with it once three considerations are taken into account. First, if RCE boundaries primarily act as material barriers for near-neutrally buoyant particles, limited contrasts are expected, when eddies are sampled close to their formation regions where the concentrations inside and outside the coherent boundary may still be similar, a scenario in which statistical significance would not be expected even if the framework holds. Second, for particles that deviate from neutral buoyancy, coherent eddies act as finite-time attractors or repellers, implying that contrasts between inner and outer regions, as well as between eddy polarities, may become more pronounced over longer timescales, which may explain why some differences were not significantly distinguished in the present dataset. Third, only a limited number of stations, particularly in the cyclone, were located within the coherent boundary, reducing statistical power, while many “outer” stations remained within the broader SLA signature and were therefore still influenced by eddy-induced flows. We therefore do not interpret the non-significant contrasts as supporting the framework; rather, we note that their occurrence is the outcome the framework would itself predict under the present sampling conditions, and that the significant contrasts observed elsewhere remain the relevant positive evidence. Overall, our observations suggest that most microplastics behave as near-neutrally buoyant particles constrained by RCE boundaries, while a fraction exhibits inertial responses consistent with non-neutral buoyancy, producing the particle-specific patterns identified in both eddies.

Both Brach et al.^25^ and Nakajima et al.^26^ sampled microplastics from the ocean surface layer, likely collecting particles that were either fully or partially submerged. Differences in sampling depth may therefore modulate the relative contribution of these particle states. Nakajima et al.^26^ sampled down to 0.4 m, likely capturing a substantial fraction of fully submerged particles, for which the inertial framework for immersed particles predicts attraction toward cyclonic RCEs for positively buoyant particles^20,46^, explaining the exceptionally high concentrations reported in their study. In contrast, the shallower sampling of Brach et al.^25^, limited to the upper 0.2 m, may increase the proportion of partially submerged particles, a regime more consistent with the surface inertial dynamics described by Beron-Vera et al.^47^, which predicts preferential accumulation in anticyclonic eddies. While this interpretation provides a physically consistent framework linking both studies, confirming the relative dominance of each dynamical regime will require dedicated investigations explicitly resolving the vertical position and buoyancy of sampled microplastics. Within this inertial perspective, our observations of fully submerged microplastics extend previous results into the water column, supporting a unified view of how mesoscale eddies modulate marine microplastic distributions. Likewise, the recent study of Ding et al.^28^ in the Western North Pacific provides further support for these particle-specific patterns, reporting larger sizes and greater diversity in microplastics within anticyclonic eddies compared to cyclonic ones in the water column. This pattern is consistent with our detection of larger fragments preferentially accumulating in the anticyclonic eddy, in line with the view that eddy polarity and its associated dynamics play a role in modulating microplastic retention below the surface. Together, these studies are complementary and highlight the role of mesoscale eddies in shaping microplastic distributions in the open ocean.

### Additional dynamics driving microplastic accumulation

Beyond mesoscale dynamics, other oceanographic processes may also influence the observed microplastic distribution. Submesoscale processes can develop within eddies and may differentially affect anticyclonic and cyclonic structures. Consistent with this interpretation, the anticyclonic eddy exhibited a weakly stratified subsurface lens, indicative of submesoscale subduction, which may partially contribute to the observed microplastic patterns.

Another important factor is the influence of the pycnocline, which can be modified by mesoscale eddies even at larger scales^67^. Stratification has been shown to affect microplastic behaviour, particularly for large fragments, through a combination of buoyancy, diffusion, and viscosity. These effects reduce sinking velocities by increasing drag and suppressing vertical motion, guiding particles along isopycnals^68,69^. This mechanism depends on a stratification length scale (L), typically ranging from 100 μm to 1 mm, such that particles larger than L are influenced, whereas smaller particles are generally unaffected. As a result, larger particles may preferentially accumulate within stratified layers^69,70^, potentially explaining the microplastic maximum observed near 150 m, primarily observed in large fragments. We acknowledge, however, that the effect of varying ambient density experienced by particles advected through multiple density layers over the depth of the eddy may be of comparable or greater importance, and the relative contribution of these mechanisms cannot be resolved from the present dataset.

By contrast, the common minima observed near 600 m across most microplastic categories in both eddies are unlikely to be directly related to mesoscale dynamics. This feature could not be resolved within the present study and warrants further investigation.

Finally, it is important to acknowledge the inherently patchy and temporally variable nature of microplastic distributions in the ocean. This variability arises from the interplay of physical, biological, and chemical processes, including wind-driven mixing, vertical advection, mesoscale and submesoscale circulation, degradation and biofouling, which alter particle buoyancy over time^16,55,71,72^. Recognising these sources of variability is therefore critical when interpreting spatial microplastic distributions, as the simultaneous influence of multiple processes makes it inherently challenging to isolate the contribution of any single mechanism from observational data.

## Conclusions

Our observations demonstrate that coherent mesoscale eddies can significantly modulate the distribution and retention of microplastics in the open ocean, extending their influence well below the surface. Distinct accumulation patterns emerged between eddy types: the anticyclone preferentially retained fragments, whereas the cyclone exhibited enhanced concentrations of fibers, highlighting particle-specific responses that can be consistent with inertial and shape-dependent dynamics.

Within both eddies, microplastic concentrations in the upper 200 m were up to threefold higher than regional background levels, indicating that mesoscale structures can substantially enhance local retention and redistribution across the upper water column. Despite differences in polarity, both eddies exhibited common vertical features, including a concentration maximum near the pycnocline (~ 150 m) and a minimum around 600 m, pointing to vertical structuring mechanisms that warrant further investigation.

Beyond individual contrasts, the persistence of enrichment and organized vertical structure across both eddy types indicates that inertial dynamics operating within rotationally coherent mesoscale eddies provide an organizing framework for microplastic distributions in the ocean.

Together, these findings identify coherent mesoscale eddies as key agents in the subsurface redistribution of microplastics in the Atlantic Ocean. By establishing an observationally grounded mechanistic link between mesoscale dynamics and subsurface microplastic accumulation, this study fills a critical gap in the oceanic plastic budget and highlights the central role of coherent circulation features in shaping the fate of plastic debris below the ocean surface.

## Supplementary Information

Below is the link to the electronic supplementary material.

Supplementary Material 1

## Data Availability

The datasets generated during and/or analysed during the current study are available from the corresponding author on reasonable request.
