# Supplementary material for: Coherent eddies redistribute microplastics across the water column
Source: Sci Rep. 2026 Sep 3;16:27672. doi: 10.1038/s41598-026-60846-7 (PMC13542135; doi:10.1038/s41598-026-60846-7)
Supplement: Supplementary file 1 [file 41598_2026_60846_MOESM1_ESM.pdf]

Coherent eddies redistribute microplastics across the water column

Álvaro Cubas[a], Eugenio Fraile-Nuez[b], Francisco J. Beron-Vera[c], Ana Molina-Rodríguez[d], Francisco Machín[a*] and Daura Vega-Moreno[d]

[a]Oceanografía Física y Geofísica Aplicada (OFYGA), IU-ECOAQUA, University of Las Palmas de Gran Canaria, 35017, Spain
[b]Centro Oceanográfico de Canarias, Instituto Español de Oceanografía (IEO), Consejo Superior de Investigaciones Científicas (CSIC), Santa Cruz de Tenerife, 38180, Spain
[c]Department of Atmospheric Sciences, Rosenstiel School of Marine, Atmospheric & Earth Science, University of Miami, Miami, FL 33149, USA
[d]OpenPLAS Group, Chemistry Department, University of Las Palmas de Gran Canaria, Las Palmas de Gran Canaria, 35017, Spain

**Correspondence to*: Francisco Machín (francisco.machin@ulpgc.es)

Oceanographic vertical sections

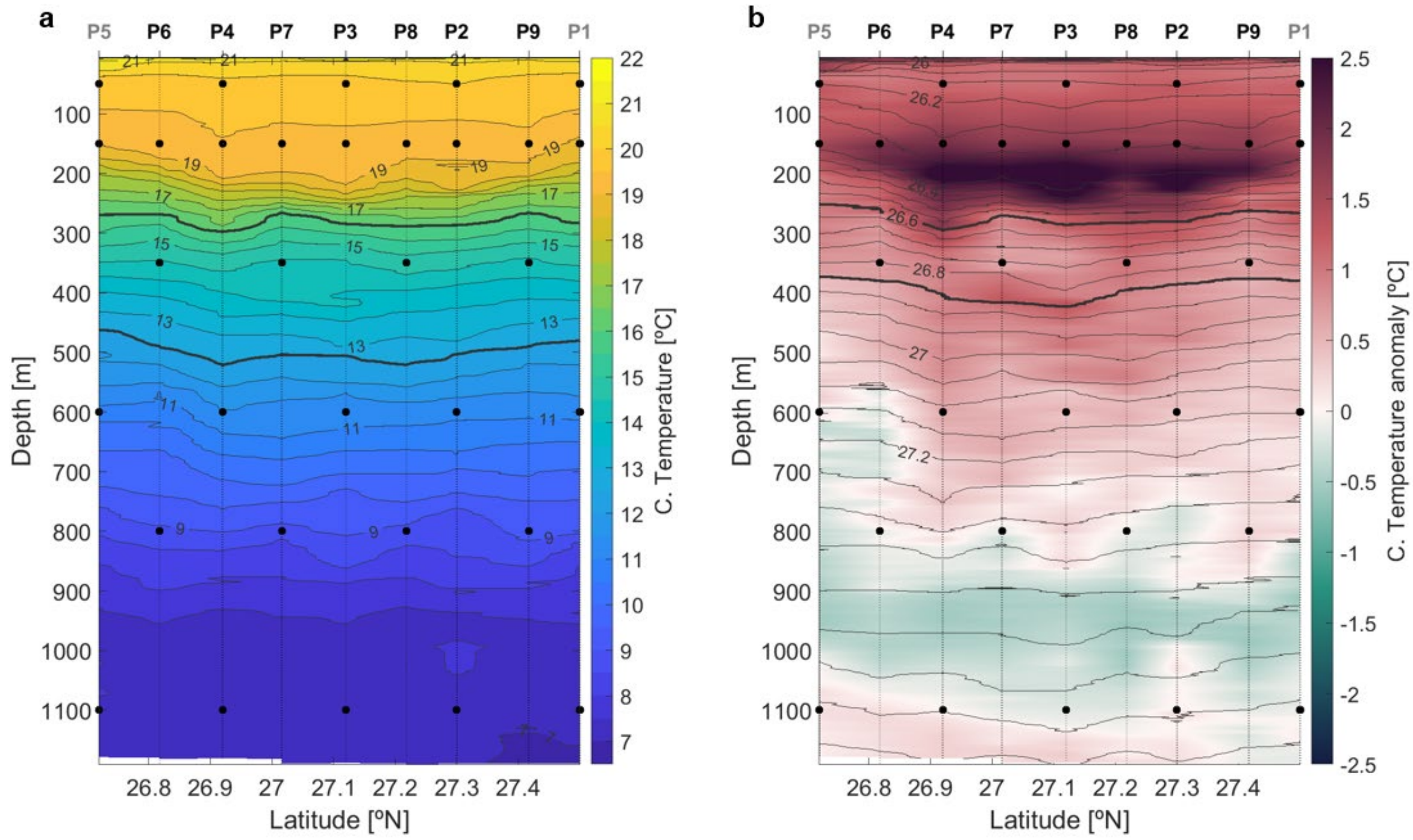

Figure S1. Vertical sections of (a) conservative temperature, and (b) its anomaly computed using the WOA23 superimposed with the isopycnals (bold lines represent the 26.85 $kg\ m^{-3}$ and 26.6 $kg\ m^{-3}$ isopycnals) for the intrathermocline eddy. The microplastic sampling depths are represented with black dots. Stations outside the coherent boundary (label in gray color) are also considered.

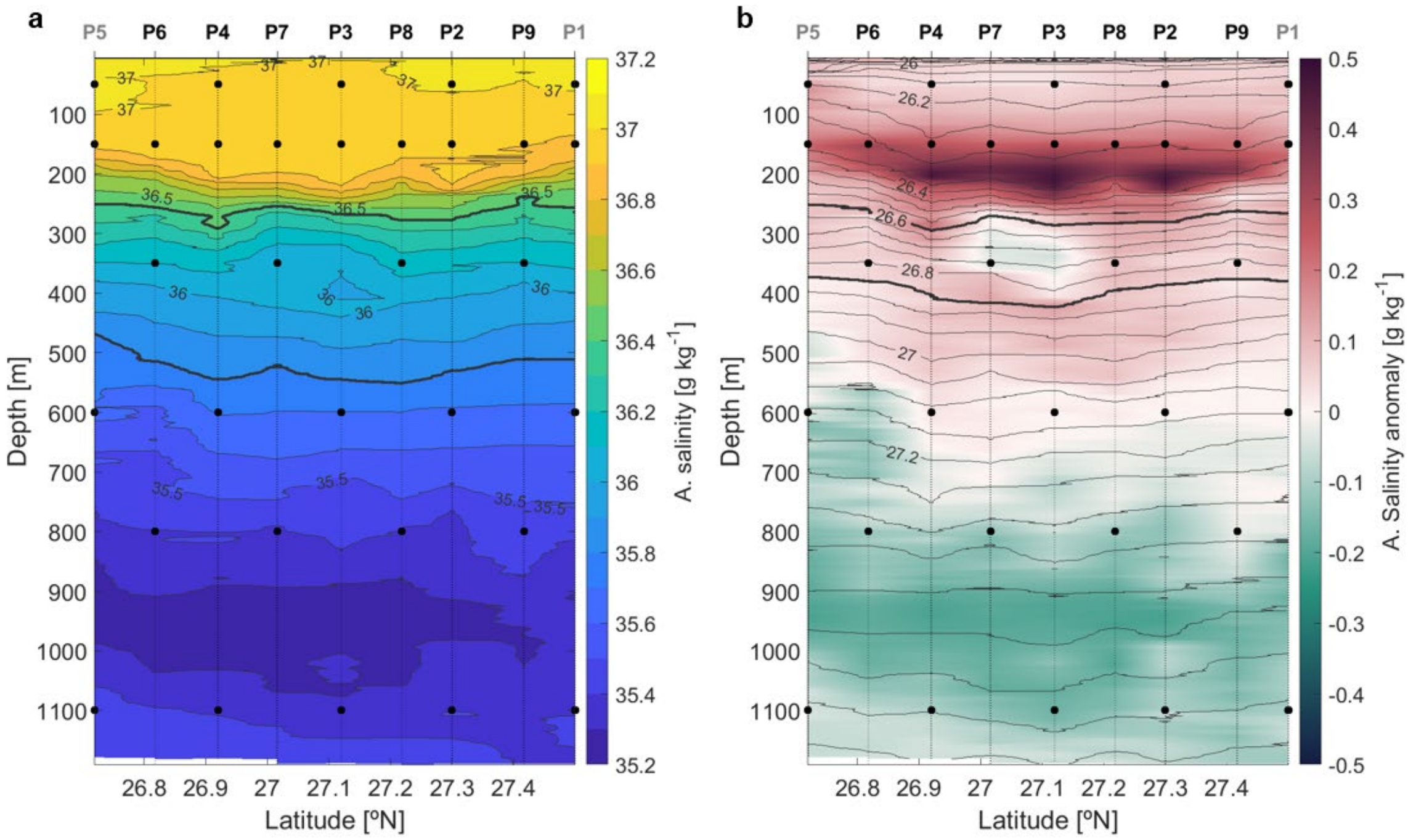

Figure S2. Vertical sections of (a) absolute salinity, the bold lines represent the 35.8 g/kg and 36.4 g/kg isohalines, and (b) its anomaly computed using the WOA23 superimposed with the isopycnals (bold lines represent the 26.85 $kg\,m^{-3}$ and 26.6 $kg\,m^{-3}$ isopycnals) for the intrathermocline eddy. The microplastic sampling depths are represented with black dots. Stations outside the coherent boundary (label in gray color) are also considered.

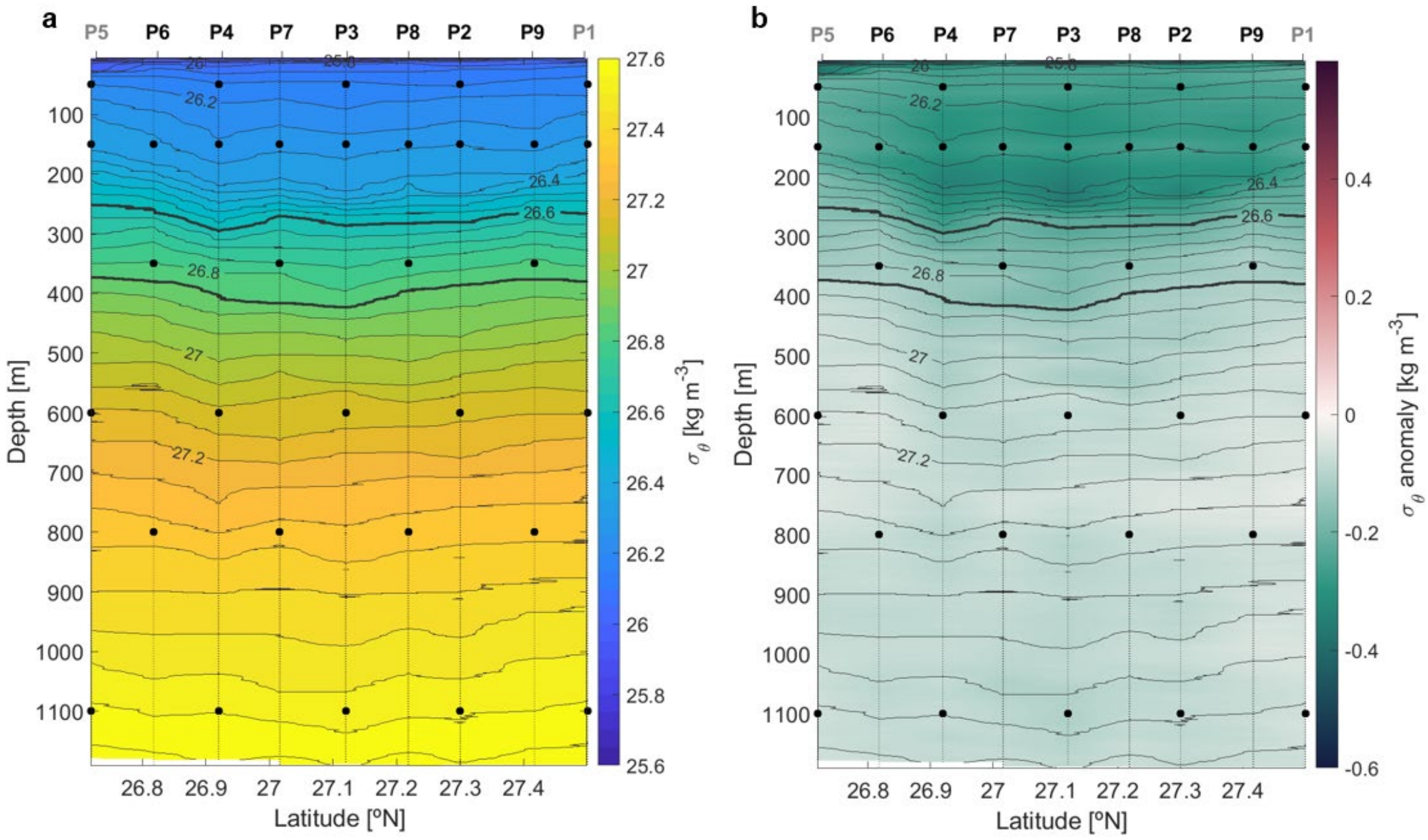

Figure S3. Vertical sections of (a) potential density anomaly, and (b) its anomaly computed using the WOA23 superimposed with the isopycnals (bold lines represent the 26.85 $kg\,m^{-3}$ and 26.6 $kg\,m^{-3}$ isopycnals) for the intrathermocline eddy. The microplastic sampling depths are represented with black dots. Stations outside the coherent boundary (label in gray color) are also considered.

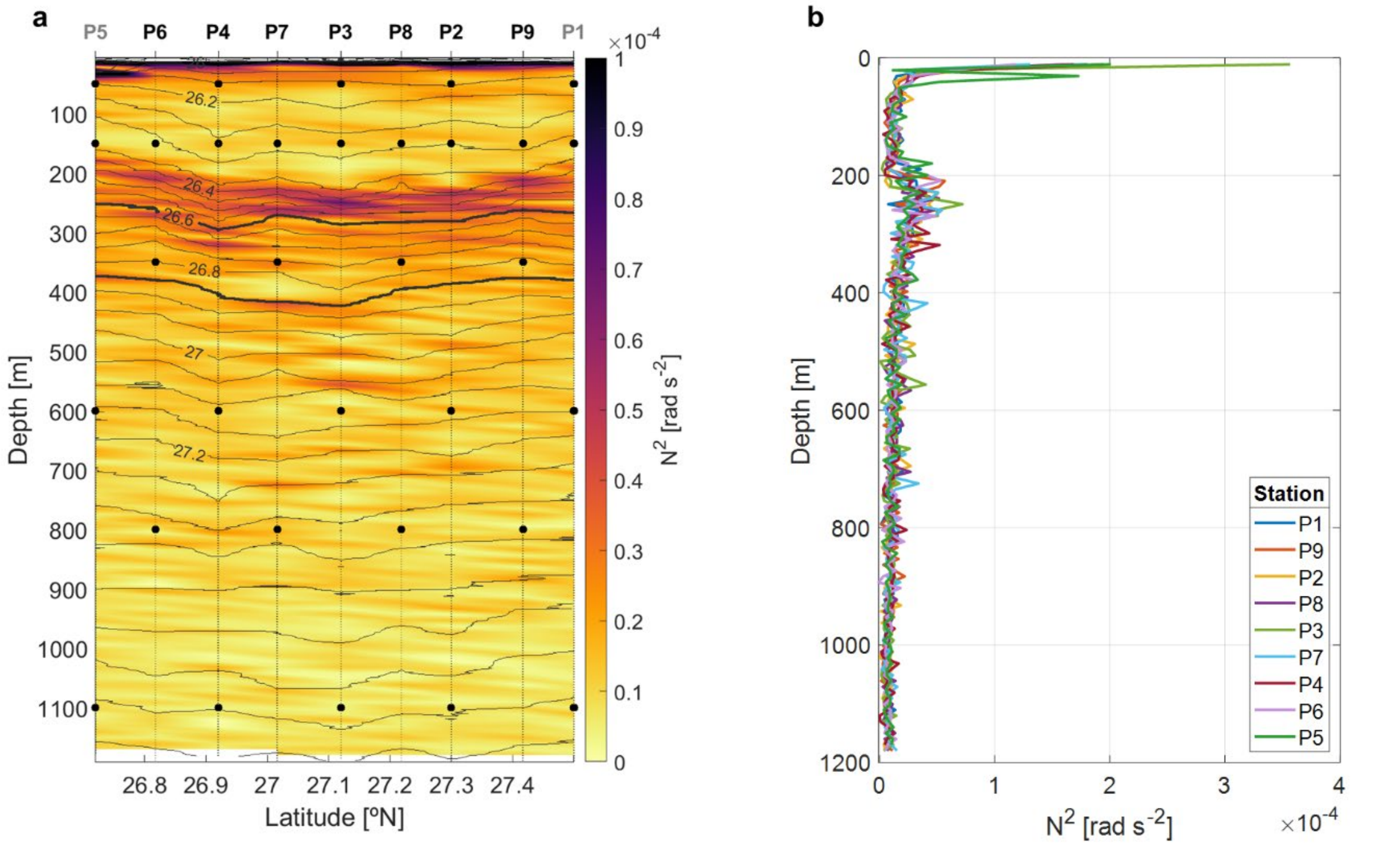

Figure S4. Representation of (a) vertical section of the brunt-väisälä frequency (bold lines represent the 26.85 $kg\ m^{-3}$ and 26.6 $kg\ m^{-3}$ isopycnals), and (b) the vertical profiles for each station for the intrathermocline eddy. The microplastic sampling depths are represented with black dots. Stations outside the coherent boundary (label in gray color) are also considered. $N^2$was computed using the TEOS-10 equation and considering grids of 10 meters in the vertical axis.

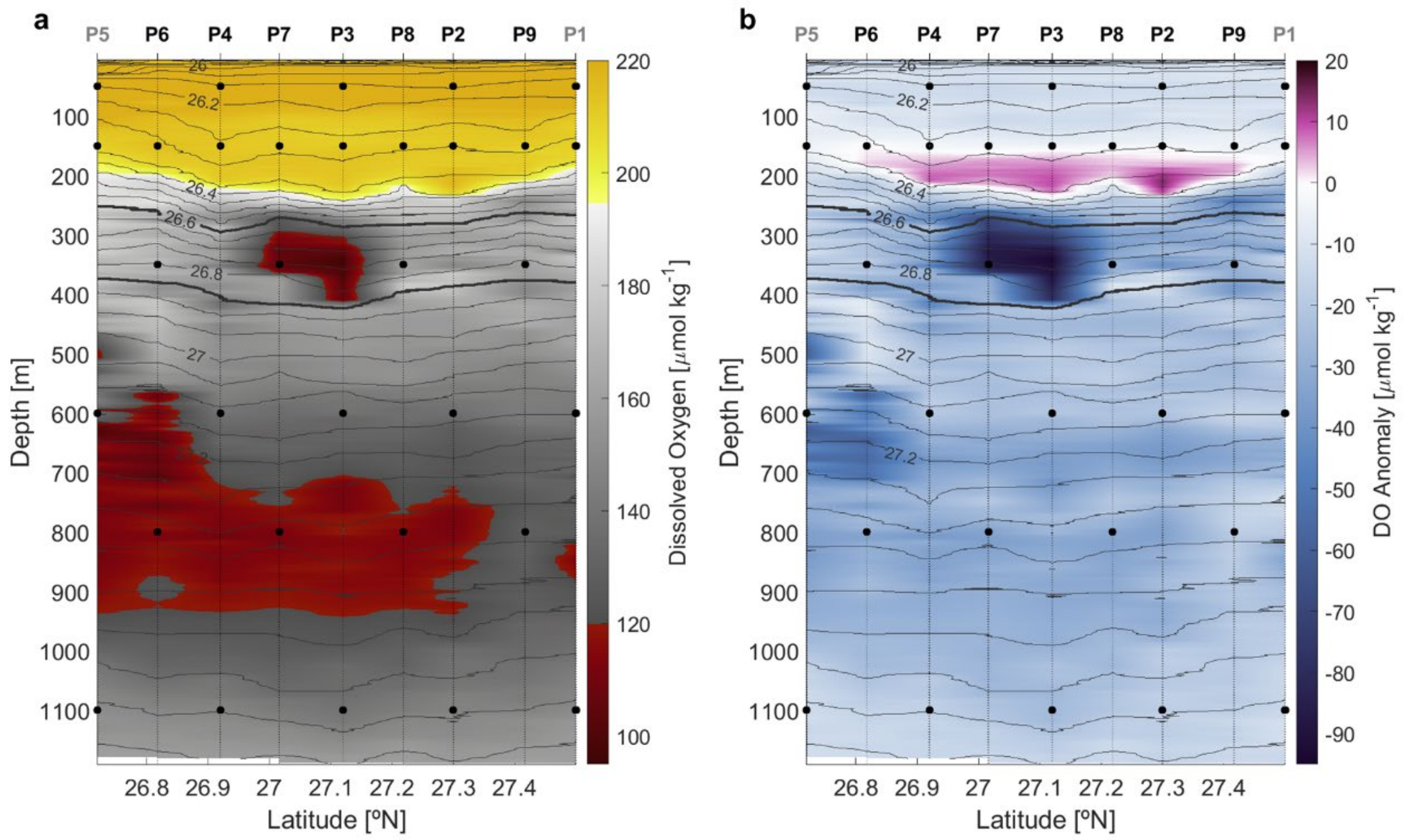

Figure S5. Vertical sections of (A) dissolved oxygen (DO) and (B) its anomaly computed using the WOA23 superimposed with the isopycnals for the intrathermocline eddy. The bold lines represent the 26.85 $kg\ m^{-3}$ and 26.6 $kg\ m^{-3}$ isopycnals. The microplastic sampling depths are represented with black dots.

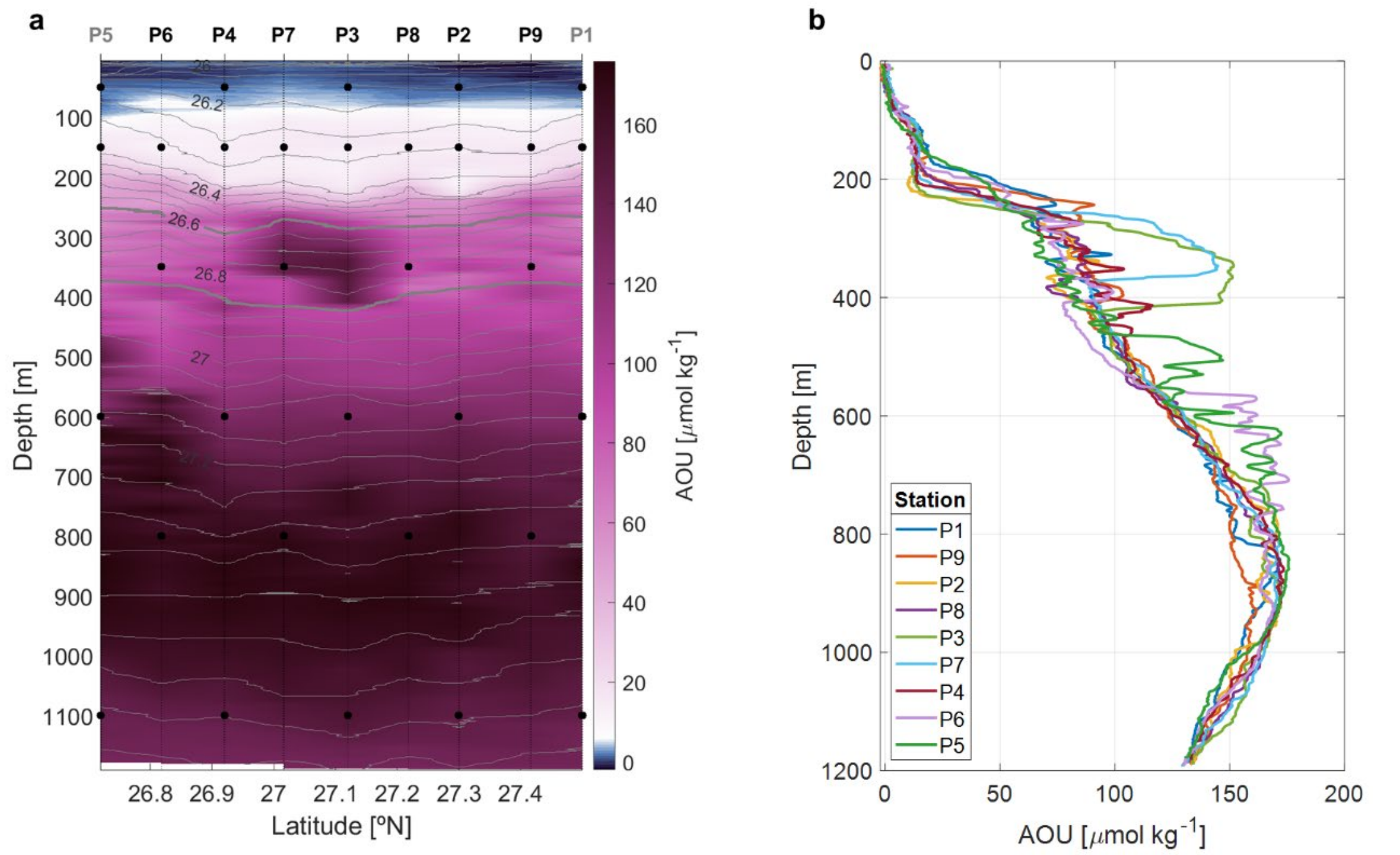

Figure S6. Representation of (a) vertical section of the Apparent Oxygen Utilization (AOU; bold lines represent the 26.85 $kg\,m^{-3}$ and 26.6 $kg\,m^{-3}$ isopycnals), and (b) the vertical profiles for each station for the intrathermocline eddy. The microplastic sampling depths are represented with black dots. Stations outside the coherent boundary (label in gray color) are also considered.

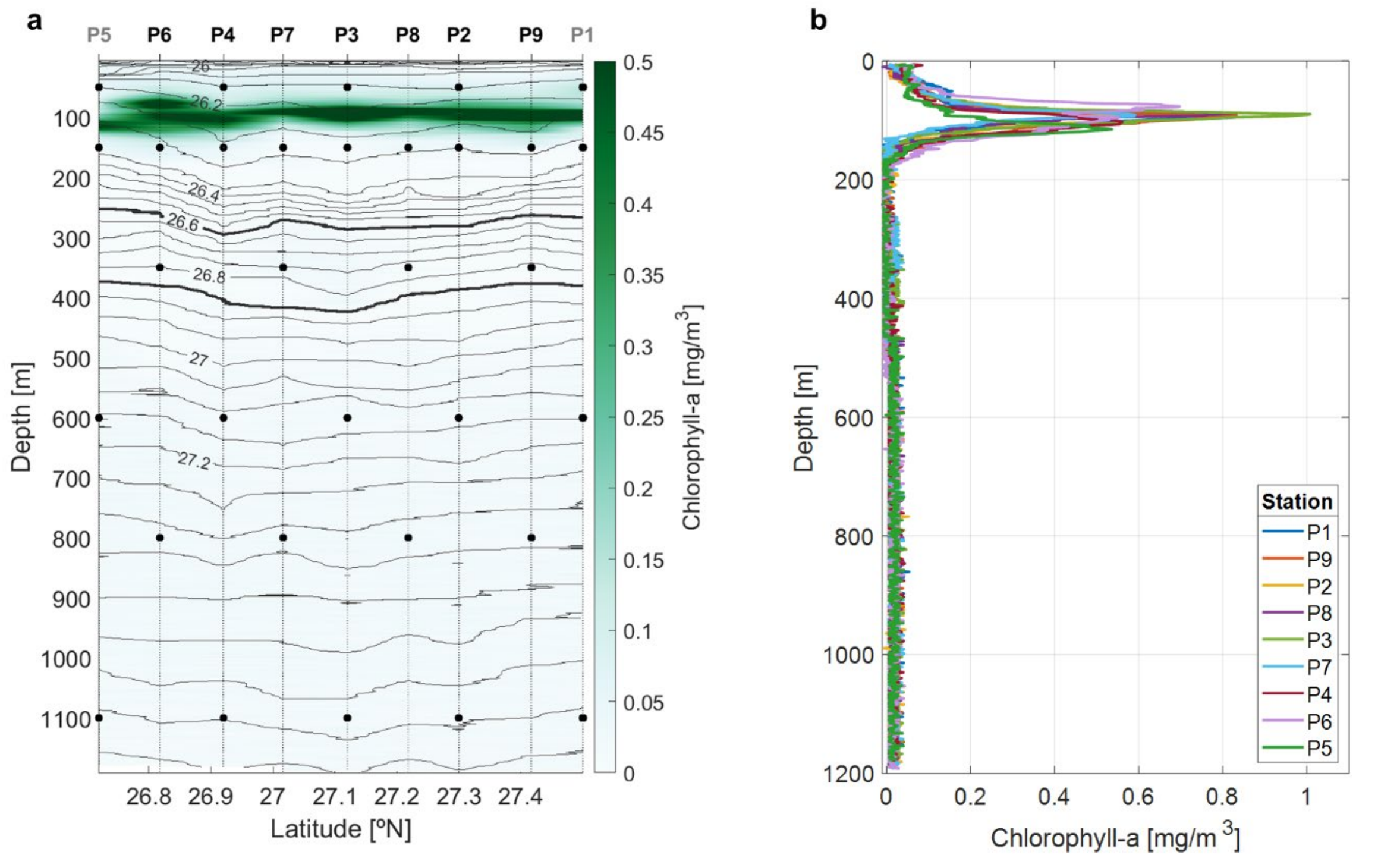

Figure S7. Representation of (a) vertical section of the Chlorophyll-a mass concentration (bold lines represent the 26.85 $kg\,m^{-3}$ and 26.6 $kg\,m^{-3}$ isopycnals), and (b) the vertical profiles for each station for the intrathermocline eddy. The microplastic sampling depths are represented with black dots. Stations outside the coherent boundary (label in gray color) are also considered.

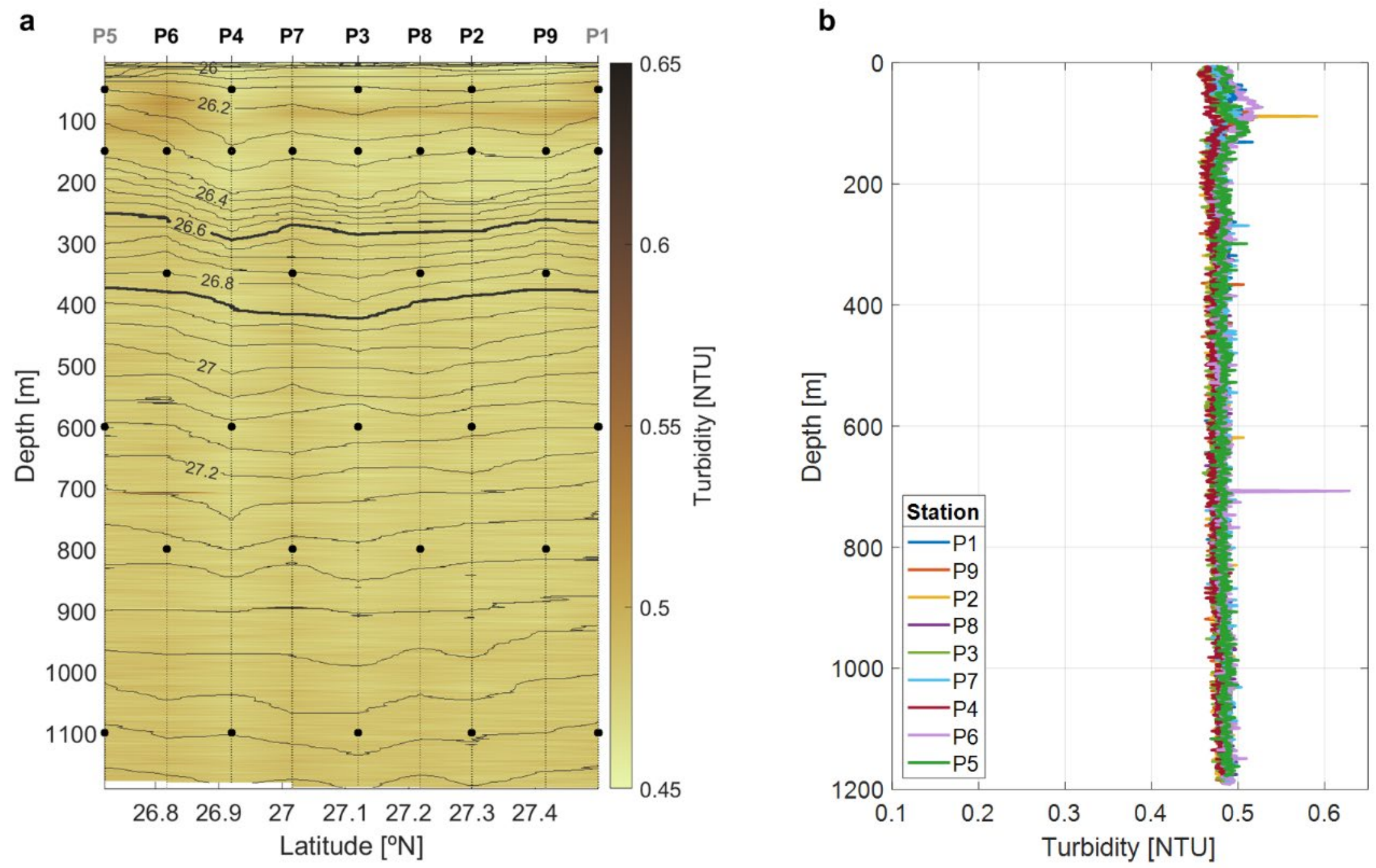

Figure S8. Representation of (a) vertical section of the Chlorophyll-a mass concentration (bold lines represent the 26.85 $kg\,m^{-3}$ and 26.6 $kg\,m^{-3}$ isopycnals), and (b) the vertical profiles for each station for the intrathermocline eddy. The microplastic sampling depths are represented with black dots. Stations outside the coherent boundary (label in gray color) are also considered.

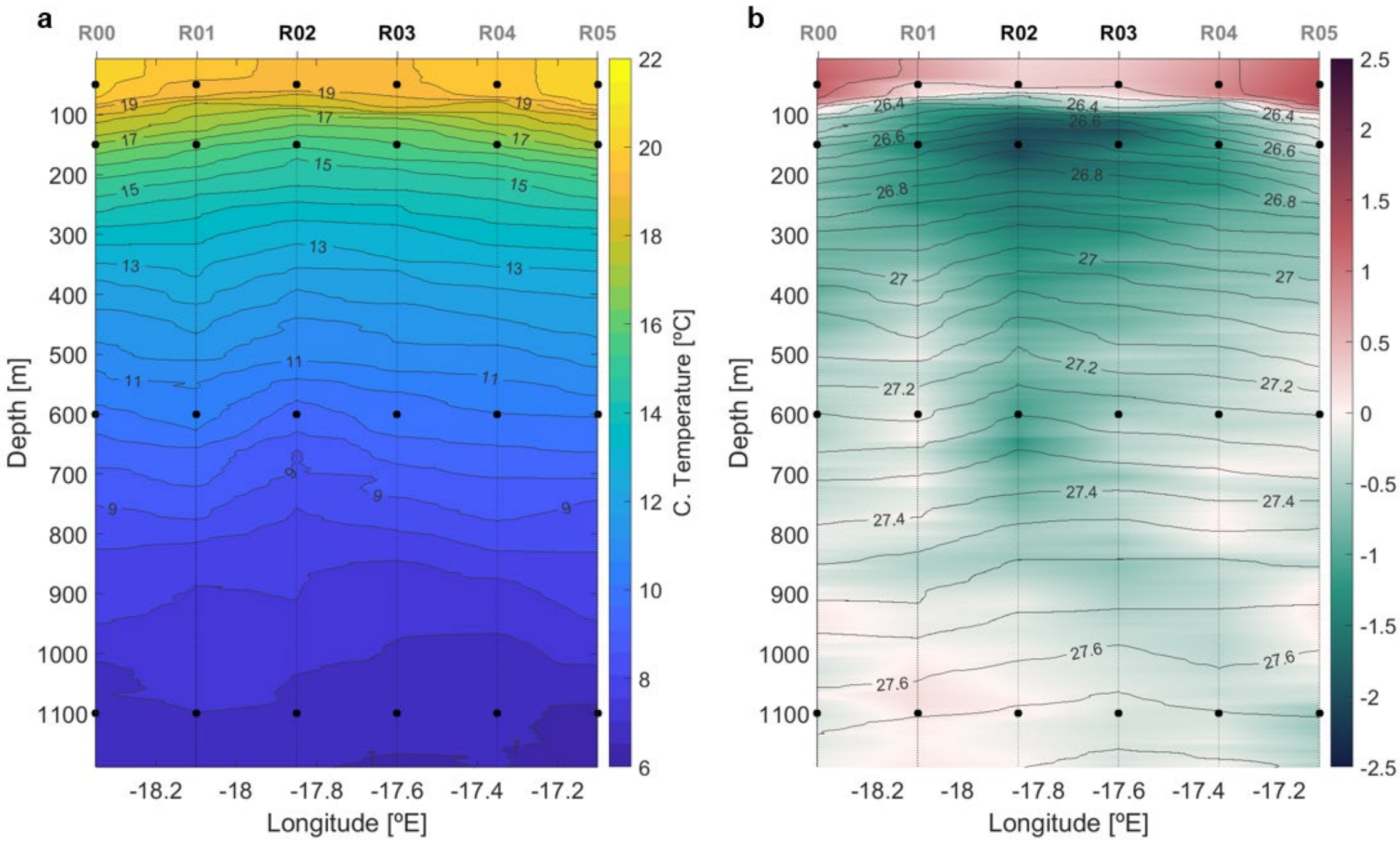

Figure S9. Vertical sections of (a) conservative temperature and (b) its anomaly computed using the WOA23 superimposed with the isopycnals for the cyclonic eddy. The microplastic sampling depths are represented with black dots. Stations outside the coherent boundary (label in gray color) are also considered.

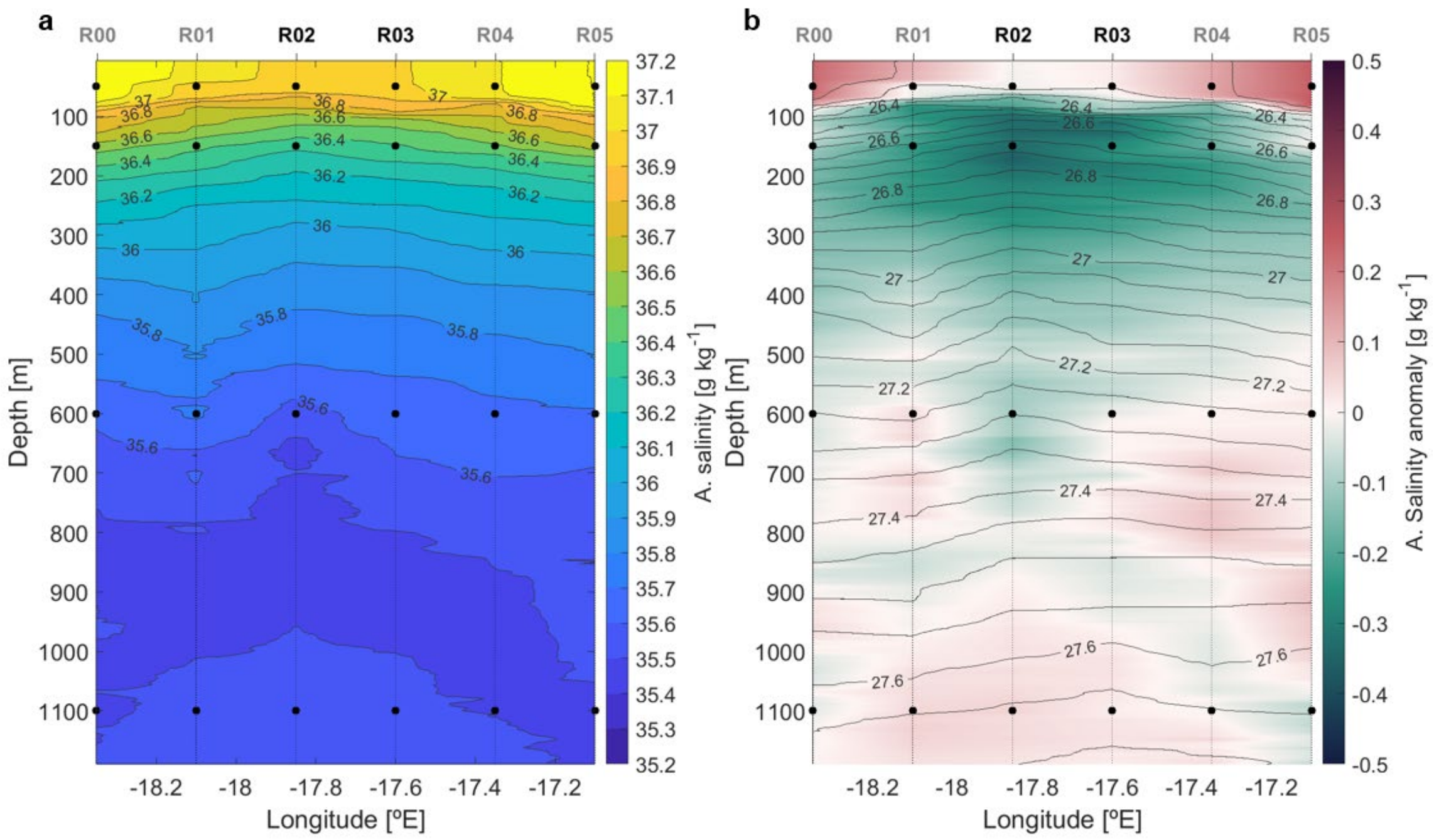

Figure S10. Vertical sections of (a) absolute salinity and (b) its anomaly computed using the WOA23 superimposed with the isopycnals for the cyclonic eddy. The microplastic sampling depths are represented with black dots. Stations outside the coherent boundary (label in gray color) are also considered.

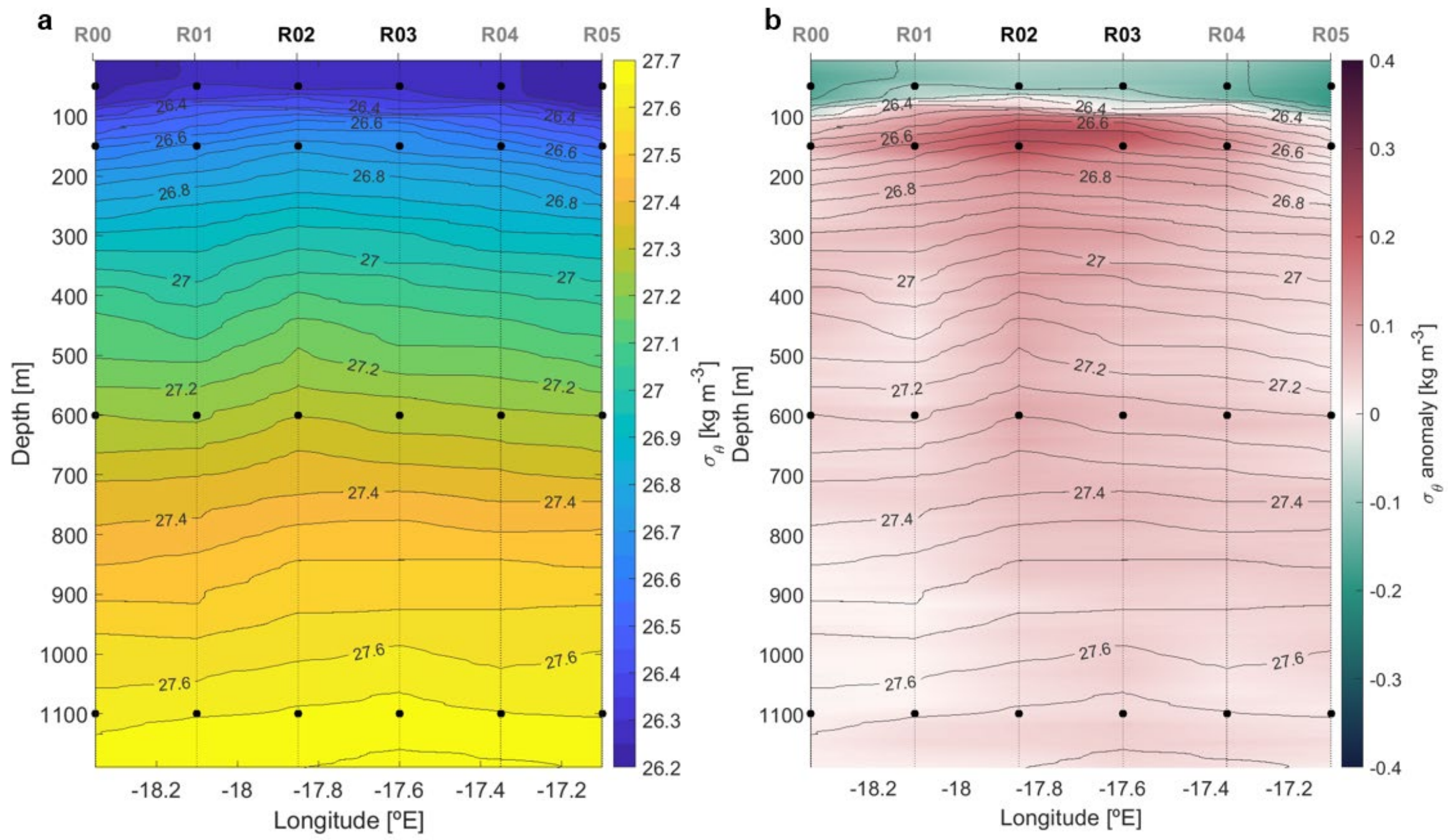

Figure S11. Vertical sections of (a) potential density anomaly and (b) its anomaly computed using the WOA23 superimposed with the isopycnals for the cyclonic eddy. The microplastic sampling depths are represented with black dots. Stations outside the coherent boundary (label in gray color) are also considered.

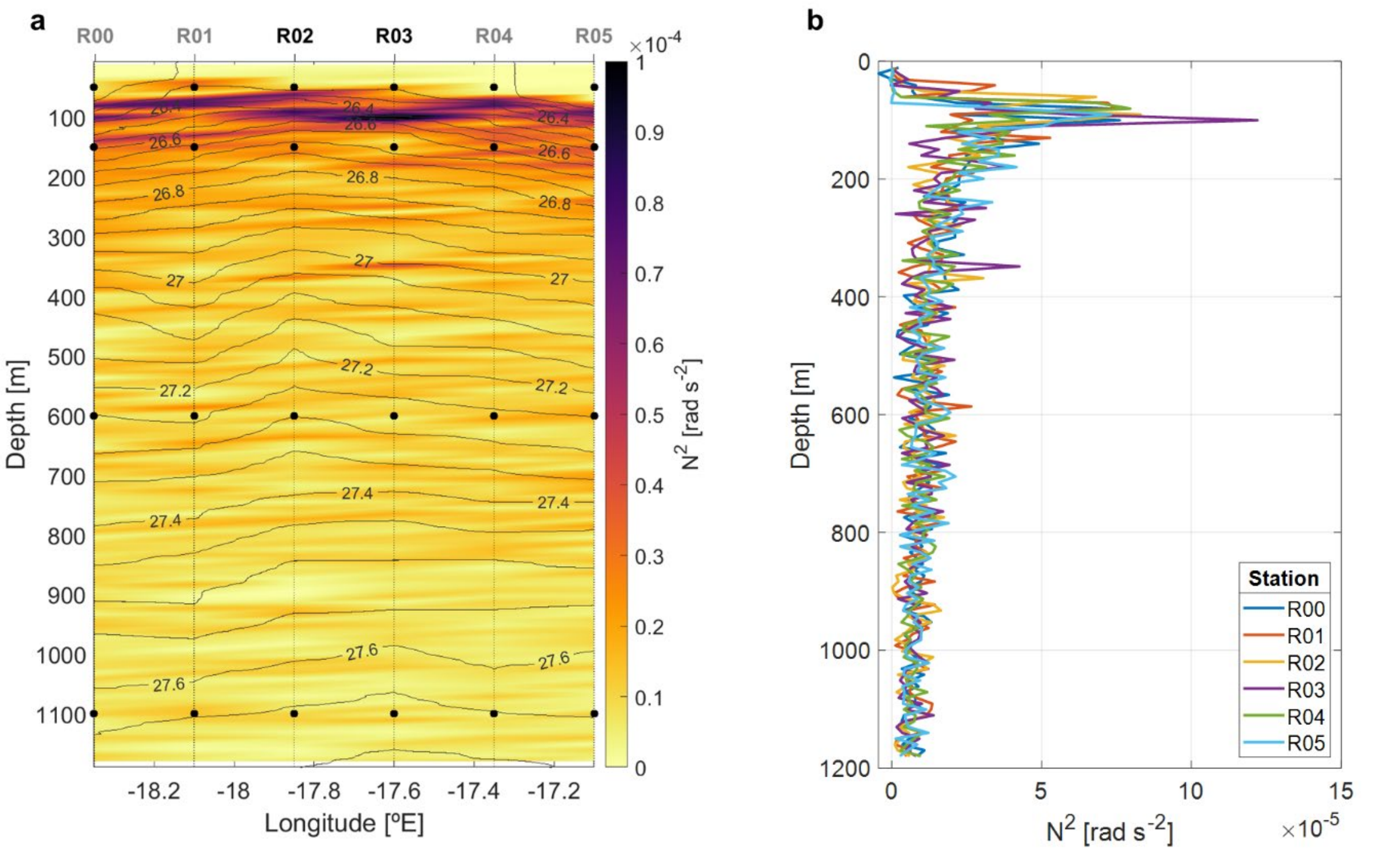

Figure S12. Representation of (a) vertical section of the brunt-väisälä frequency and (b) the vertical profiles for each station for the cyclonic eddy. The microplastic sampling depths are represented with black dots. Stations outside the coherent boundary (label in gray color) are also considered. N^2 was computed using the TEOS-10 equation and considering grids of 10 meters in the vertical axis.

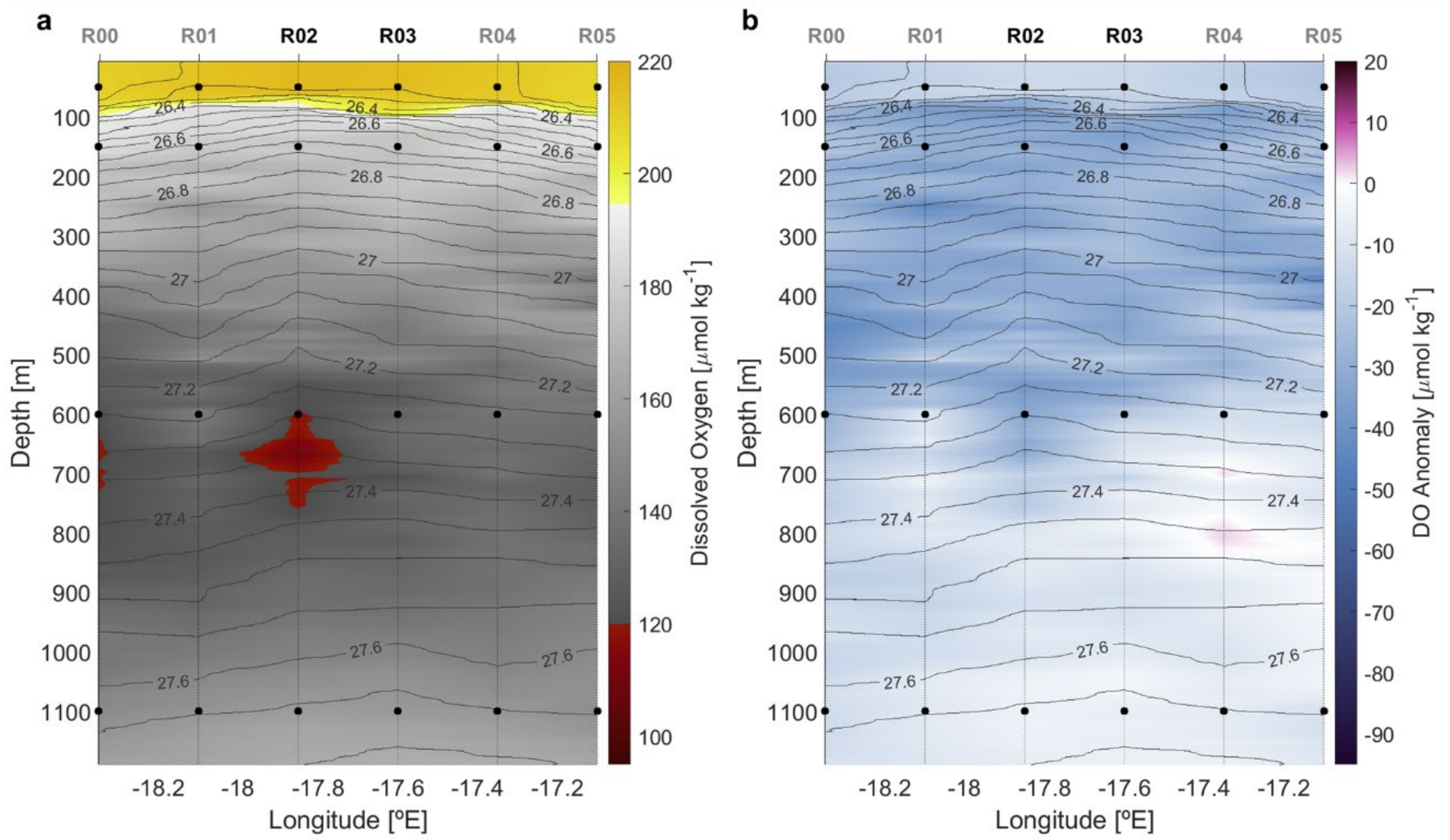

Figure S13. Vertical sections of (a) dissolved oxygen and (b) its anomaly computed using the WOA23 superimposed with the isopycnals for the cyclonic eddy. The microplastic sampling depths are represented with black dots. Stations outside the coherent boundary (label in gray color) are also considered.

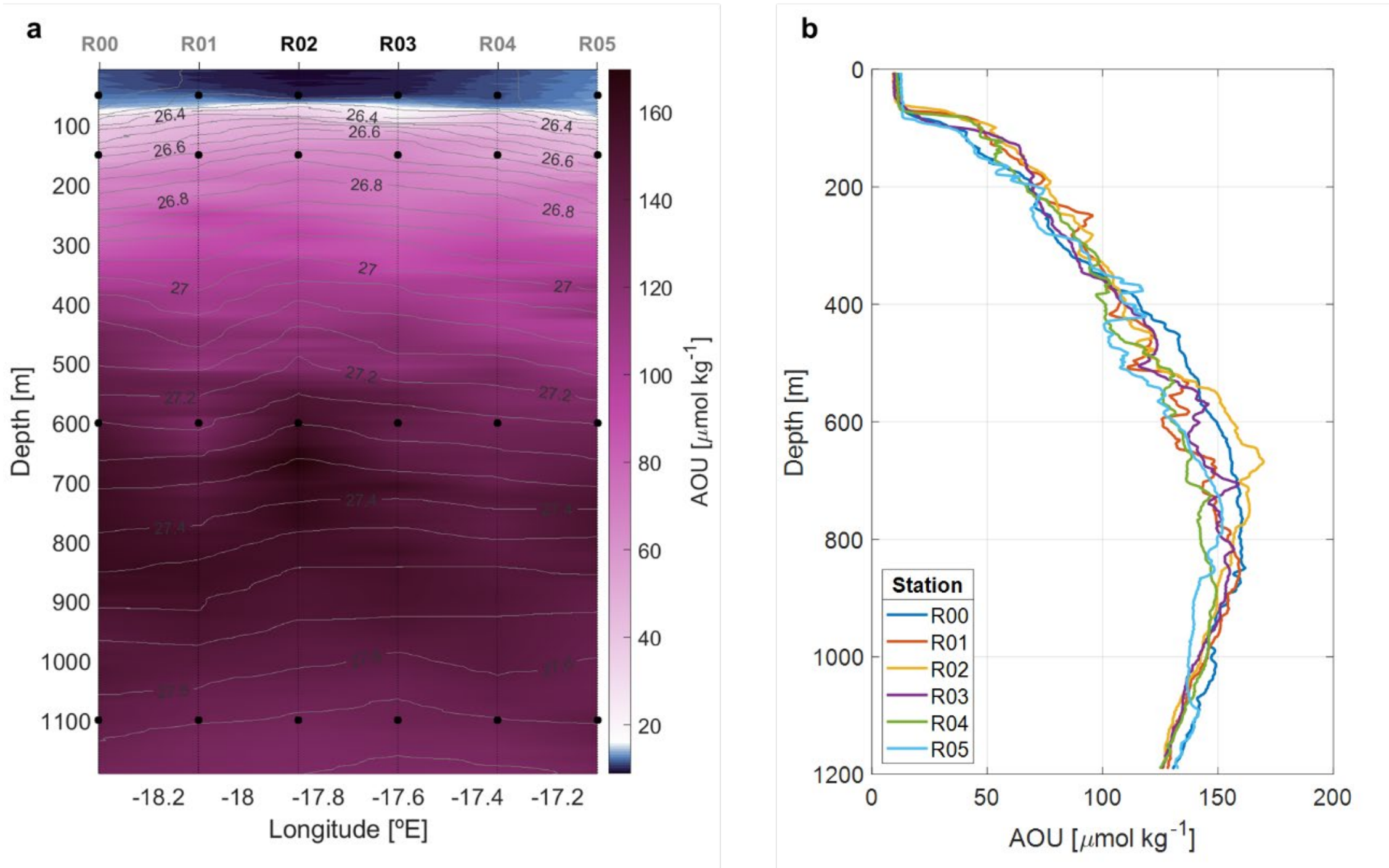

Figure S14. Representation of (a) vertical section of the Apparent Oxygen Utilization (AOU)and (b) the vertical profiles for each station for the cyclonic eddy. The microplastic sampling depths are represented with black dots. Stations outside the coherent boundary (label in gray color) are also considered.

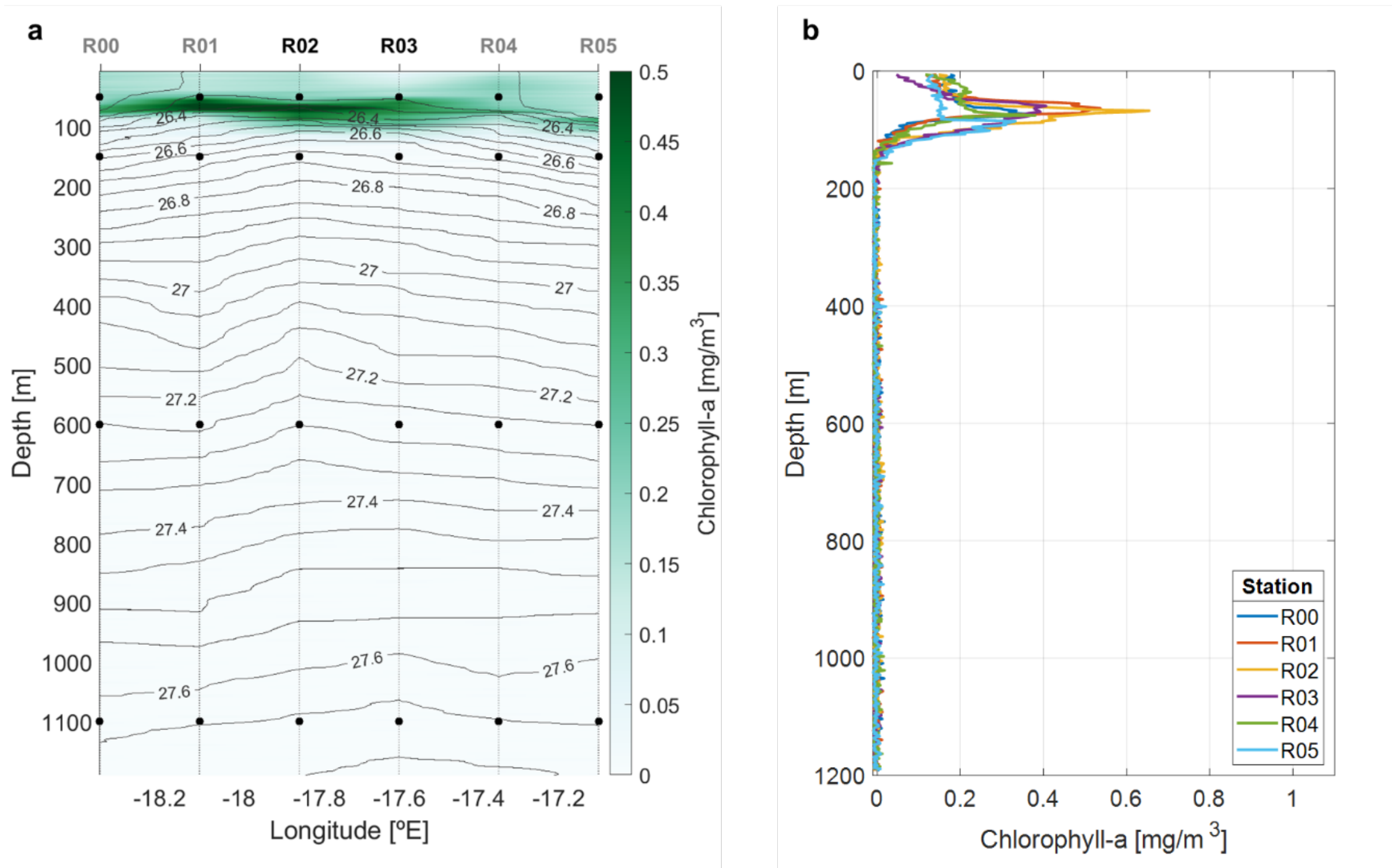

Figure S15. Representation of (a) vertical section of Chlorophyll-a mass concentration and (b) the vertical profiles for each station for the cyclonic eddy. The microplastic sampling depths are represented with black dots. Stations outside the coherent boundary (label in gray color) are also considered.

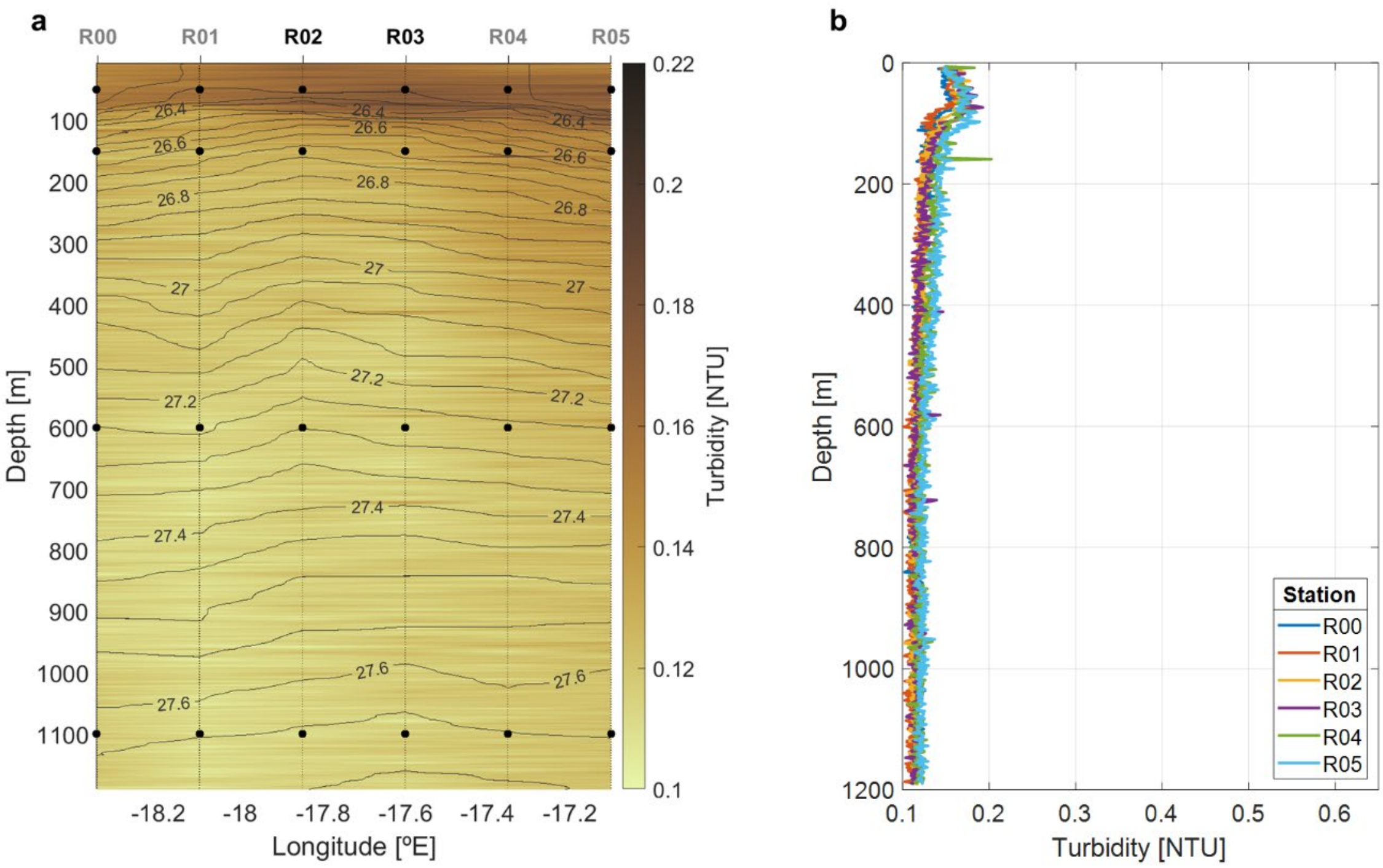

Figure S16. Representation of (a) vertical section of turbidity and (b) the vertical profiles for each station for the cyclonic eddy. The microplastic sampling depths are represented with black dots. Stations outside the coherent boundary (label in gray color) are also considered.

Inside-outside statistical comparison of microplastics

Table S1. Statistical comparison of microplastic concentrations inside and outside coherent eddy contours.

Eddy	Particle type	n (inside)	n (outside)	Mean inside	Mean outside	Welch t(df)	p (Welch)	Mann–Whitney W	p (MW)
Anticyclonic	Fibers	15	6	2.74	4.24	t(6.23)=2.63	0.0376	77.5	0.0127
Anticyclonic	Large fragments	15	6	5.82	4.69	t(18.38)=−0.67	0.5093	46.0	0.9690
Anticyclonic	Small fragments	15	6	53.01	60.64	t(9.40)=0.39	0.7055	55.0	0.4596
Cyclonic	Fibers	6	12	5.72	6.48	t(15.88)=0.63	0.5370	35.5	1.0000
Cyclonic	Large fragments	6	12	0.58	0.97	t(15.58)=2.20	0.0436	55.5	0.0750
Cyclonic	Small fragments	6	12	43.44	64.77	t(13.96)=2.26	0.0402	57.0	0.0546

Mean microplastic concentration used for the R_r computation.

Table S2. Mean microplastic concentration in the upper 200 m inside coherent eddy contours

Eddy	Particle type	Mean concentration (items/L)
Anticyclonic	Fibers	3.14
Anticyclonic	Large fragments	7.78
Anticyclonic	Small fragments	67.8
Cyclonic	Fibers	5.63
Cyclonic	Large fragments	0.60
Cyclonic	Small fragments	40.72
